# Ndufs1 Deficiency Aggravates the Mitochondrial Membrane Potential Dysfunction in Pressure Overload-Induced Myocardial Hypertrophy

**DOI:** 10.1155/2021/5545261

**Published:** 2021-03-03

**Authors:** Rongjun Zou, Jun Tao, Junxiong Qiu, Wanting Shi, Minghui Zou, Weidan Chen, Wenlei Li, Na Zhou, Shaoli Wang, Li Ma, Xinxin Chen

**Affiliations:** ^1^Heart Center, Guangdong Provincial Key Laboratory of Research in Structural Birth Defect Disease, Guangzhou Women and Children's Medical Center, Guangzhou Medical University, Guangzhou 510623, China; ^2^Department of Cardiovascular Surgery, Sun Yat-sen Memorial Hospital, Sun Yat-sen University, Guangzhou, Guangdong 510120, China; ^3^Department of Paediatrics, Guangdong Provincial Key Laboratory of Research in Structural Birth Defect Disease, Guangzhou Women and Children's Medical Center, Guangzhou Medical University, Guangzhou 510623, China; ^4^Department of Surgical Nursing, Guangzhou Women and Children's Medical Center, Guangzhou Medical University, Guangzhou 510623, China

## Abstract

Mitochondrial dysfunction has been suggested to be the key factor in the development and progression of cardiac hypertrophy. The onset of mitochondrial dysfunction and the mechanisms underlying the development of cardiac hypertrophy (CH) are incompletely understood. The present study is based on the use of multiple bioinformatics analyses for the organization and analysis of scRNA-seq and microarray datasets from a transverse aortic constriction (TAC) model to examine the potential role of mitochondrial dysfunction in the pathophysiology of CH. The results showed that NADH:ubiquinone oxidoreductase core subunit S1- (Ndufs1-) dependent mitochondrial dysfunction plays a key role in pressure overload-induced CH. Furthermore, *in vivo* animal studies using a TAC mouse model of CH showed that Ndufs1 expression was significantly downregulated in hypertrophic heart tissue compared to that in normal controls. In an *in vitro* model of angiotensin II- (Ang II-) induced cardiomyocyte hypertrophy, Ang II treatment significantly downregulated the expression of Ndufs1 in cardiomyocytes. *In vitro* mechanistic studies showed that Ndufs1 knockdown induced CH; decreased the mitochondrial DNA content, mitochondrial membrane potential (MMP), and mitochondrial mass; and increased the production of mitochondrial reactive oxygen species (ROS) in cardiomyocytes. On the other hand, Ang II treatment upregulated the expression levels of atrial natriuretic peptide, brain natriuretic peptide, and myosin heavy chain beta; decreased the mitochondrial DNA content, MMP, and mitochondrial mass; and increased mitochondrial ROS production in cardiomyocytes. The Ang II-mediated effects were significantly attenuated by overexpression of Ndufs1 in rat cardiomyocytes. In conclusion, our results demonstrate downregulation of Ndufs1 in hypertrophic heart tissue, and the results of mechanistic studies suggest that Ndufs1 deficiency may cause mitochondrial dysfunction in cardiomyocytes, which may be associated with the development and progression of CH.

## 1. Introduction

Cardiac hypertrophy (CH) is a pathophysiological response characterized by increased thickness of the ventricular wall, greater myocardial cell volume, and enhanced myocardial contractility during the early stage of overload pressure [1]. Primarily, CH is the compensatory response for preservation of cardiac function; however, persistent CH is often associated with disturbed energy metabolism, deteriorated cardiac function, and interstitial fibrosis, which will eventually progress into heart failure [2, 3]. Heart failure caused by CH has been shown to be an independent risk factor for various cardiovascular diseases [4, 5]. To date, the pathophysiology underlying the progression of myocardial hypertrophy remains elusive. Thus, determination of potential molecular mechanisms is necessary for identification of novel and effective therapies to attenuate myocardial hypertrophy.

The contraction and relaxation of cardiomyocytes require a sufficient energy supply to meet the workload demand, and mitochondria are important primary organelles for energy production in cardiomyocytes [6–8]. Mitochondrial dysfunction was shown to be closely associated with the development of heart failure [9–11]. Under pathological conditions of CH, the activities of ATP synthase and mitochondrial oxidative phosphorylation complex are attenuated, which results in reduced production of ATP [12, 13]. Moreover, attenuated mitochondrial dynamics, reduced mitochondrial volume, and abnormal mitochondrial morphology were detected in cardiomyocytes in CH [14, 15]. Mitochondrial dysfunction was shown to increase the production of reactive oxygen species (ROS) via impaired electron transport chains, which can lead to increased oxidative stress and decreased energy production in cardiomyocytes [9, 16, 17]. Thus, restoration of impaired mitochondrial functions will provide novel strategies to attenuate the progression of CH. NADH:ubiquinone oxidoreductase core subunit S1 (Ndufs1) is one of the core subunits of mitochondrial complex I that regulates mitochondrial oxidative phosphorylation and ROS production [18–20]. However, the detailed role of Ndufs1 in the pathophysiology of CH is largely unknown.

In the present study, we initially demonstrated the deregulation of Ndufs1 in heart tissue of mice with CH by analyzing the GSE95140 scRNA-seq dataset. The expression of Ndusf1 was confirmed in heart tissue in a mouse model of CH. Furthermore, *in vitro* studies determined the molecular mechanisms of Ndusf1-mediated CH. The present study may provide novel insight into the role of Ndusf1 in the pathophysiology of CH.

## 2. Materials and Methods

### 2.1. Analysis of scRNA-seq and Microarray Datasets

RNA sequencing data for single cardiomyocytes were downloaded from the GSE95140 dataset of the GEO database [21]. This dataset is based on the GPL17021 platform and contains 396 single-cardiomyocyte transcriptomes of mice after transverse aortic constriction (TAC) or sham operation assayed on day 3 (D3), week 1 (W1), week 2 (W2), week 4 (W4), and week 8 (W8). The expression in each cell was detected by using the “DropletUtils” package. Gene expression in the cells was calculated using the “QC-Metrics” function in the “scater” package [22]. Ribosomal genes ≥ 10% and mitochondrial genes ≤ 5% were used for subsequent filtering. After filtering, the expression matrix of each sample was normalized by using the “NormalizeData” function of the “Seurat” package (version 3.0) [23]. The genes with the most pronounced differences between the cells were selected using the “FindVariableFeatures” function of the “Seurat” package. The “ScaleData” function was used to convert the expression data to linear scale. Then, principal component analysis (PCA) was performed using the “RunPCA” function of the “Seurat” package. Principal components (PCs) with standard deviations > 70% were selected. “RunUMAP” of the “Seurat” package was employed to perform UMAP dimensionality reduction analysis. The “FindAllMarkers” function of the “Seurat” package was used to define the criteria for identification of differentially expressed genes (DEGs) as follows: cell population expression ratio > 0.25, log | fold change (FC) | >0.25, and *p* ≤ 0.05.

The differentially expressed genes were validated using the GSE24454 microarray dataset. In this dataset, mice were sacrificed 4 weeks after aortic banding (AB) or sham procedure (sAB) and subsequent debanding, including banding and subsequent debanding (DB3) or sham procedure and subsequent debanding (sDB3); the data were obtained at various time points up to day 3 [24]. Thus, the CEL raw data and corresponding annotation platform file were downloaded and preprocessed by background adjustment, normalization, probe summarization, and log_2_ transformation of the expression values using the “Affy” package in R.

### 2.2. Gene Ontology (GO) Term Enrichment Analysis

GO enrichment analysis was performed using the “clusterProfiler” package in R [25]. Notably, the major GO terms of DE genes in biological processes, molecular functions, cellular components, and pathways were evaluated. The Benjamini-Hochberg method was used to adjust the original *p* values. The GO terms corresponding to the DE genes were enriched with the threshold of correction *p* value < 0.05. Additionally, the enrichment analyses of the biological processes of the hub genes were carried out with the ToppGene tool (https://toppgene.cchmc.org/), which is a web-based analytic tool used for functional enrichment analysis of the gene lists [26]. Additionally, the cellular compartment-specific protein-protein interaction network was constructed by the ComPPI database (https://comppi.linkgroup.hu/) [27].

### 2.3. Gene Set Enrichment Analysis (GSEA)

GSEA was used to assess the Kyoto Encyclopedia of Genes and Genomes (KEGG) maps involved in TCA-induced CH development based on time series analysis [28]. Initially, the Kolmogorov-Smirnov method was used to determine the enrichment score (ES); then, the statistical significance of ES was assessed using the empirical phenotype replacement test procedure. The enrichment score (NES) was derived by normalization of ES for each gene set. The false discovery rate (FDR) of each NES was determined.

### 2.4. Gene Set Variation Analysis (GSVA)

The GSVA package of R was used to analyze the activation of the gene sets by unsupervised and nonparametric scoring calculations [29]. The hub pathway-related scores were calculated by the GSVA method in each cell based on the transcription expression matrix after assigning various groups in the TAC model. Significant differences in GSVA scores between various groups were assessed by one-way ANOVA.

### 2.5. Animals and Surgical Intervention

All animal experiments were approved by the Animal Ethics Committee of Sun Yat-sen University (SYSU-IACUC-2020-000469). Sixteen male C57BL/6 mice (8 weeks old) were purchased from Sun Yat-sen University, and the mice were randomly divided into two groups, including the sham (*n* = 8) and TAC groups (*n* = 8). Before operation, the animals were anaesthetized by intraperitoneal injection with 100 mg/kg ketamine + 5 mg/kg xylazine. After the animals reached general anesthesia, a small incision was made in the second intercostal space at the left upper sternal border to open the chest cavity, and the animals were subjected to respiratory ventilation. After exposure of the aortic arch, TAC was performed by tying a 7-0 nylon suture ligature against a 27-gauge needle between the left common carotid artery and the brachiocephalic artery. Then, the needle was quickly retracted to complete the partial constriction procedure. Sham-operated mice were subjected to the same surgical procedures without transverse aortic constriction. The chest was closed with 5-0 nonabsorbable sutures. Postoperatively, the animals were subcutaneously injected with 1.0 mg/kg buprenorphine to relieve postoperative pain every 12 h for 3 consecutive days. The mice were closely monitored every day for body weight and any signs of labored breathing or postoperative pain.

### 2.6. Echocardiography

Four weeks after ascending TAC operation, the animals from the sham and TAC groups were subjected to echocardiography examination. Briefly, the mice were anaesthetized by 3% isoflurane using an anesthesia machine. The hair on the left chest was carefully removed, and cardiac geometry was determined from the parasternal long axis view with a probe frequency of 30 MHz using a small animal color ultrasonic diagnostic apparatus (Vevo 2100, VisualSonics, Toronto, Canada). The images of the left ventricular area were captured using M-type echocardiography. The interventricular septum (IVS) thickness and left ventricular posterior wall (LVPW) thickness were measured.

### 2.7. Evaluation of Cardiac Index

After assessment by echocardiography, the animals were sacrificed by an overdose of 5% isoflurane. The heart was immediately dissected and rinsed with ice-cold saline to remove blood clots. After draining the heart tissue on sterile paper, the whole weight of the heart was measured using a digital balance. The left ventricular weight (LVW) was determined by removing the atrium and right ventricle from the whole heart. The heart mass index (HMI) and left ventricular mass index (LVMI) were calculated as follows: HMI = LVW/body weight; LVMI = LVW/body weight. The length of the medial malleolar distance on the right hindlimb to the tibial plateau edge was defined as the tibia length (TL). The ratios of LVW to TL were used as an index of cardiac hypertrophy.

### 2.8. Hematoxylin and Eosin (H&E) Staining

After animals were sacrificed by an overdose of 5% isoflurane, a part of the heart tissue was fixed with 4% paraformaldehyde and embedded in paraffin. The paraffin-embedded heart tissue was sectioned into 5 *μ*m sections and stained by hematoxylin and eosin. The stained sections were examined under a light microscope (Nikon, Tokyo, Japan).

### 2.9. Transmission Electron Microscopy (TEM)

The mitochondria in the heart tissue were evaluated by TEM. Briefly, the heart tissue was sectioned into 1 mm^3^ pieces, which were fixed with 4% glutaraldehyde and 1% osmic acid. Then, the tissue was dehydrated with acetone, embedded in Epon 821, and cut into 70 nm sections. Then, the sections were double stained with uranyl acetate and lead citrate. The mitochondria were examined using TEM (JEM-1230, Tokyo, Japan). Mitochondrial volume and mitochondrial number were evaluated based on the TEM images.

### 2.10. Rat Cardiomyocyte Culture

Neonatal Sprague-Dawley rats (1-2 days old) were sacrificed by cervical dislocation, and the heart was immediately dissected under sterile conditions. Ventricular tissue was isolated from the atria and digested in Hanks balanced salt solution containing 0.25% trypsin (Sigma-Aldrich, St. Louis, USA) at 37°C for 5 min, and the digestion cycle was repeated 10 times. After digestion, the supernatants were pooled and mixed with an equal volume of DMEM supplemented with 10% fetal bovine serum (FBS; Thermo Fisher Scientific, Waltham, USA). After centrifugation at 1,000 × *g* for 5 min, the supernatant was discarded, and the cell pellet was resuspended in DMEM supplemented with 10% FBS. After incubation for 4 h at 37°C in a humidified 5% CO_2_ incubator, cardiomyocytes were collected from the medium. Cardiac fibroblasts adhered to the walls of the dishes. Cardiomyocytes were cultured in 6-well plates for 24 h and in fresh DMEM supplemented with 10% FBS for 2-3 days before *in vitro* assays.

### 2.11. Construction of the Ndusf1 siRNA and Overexpression Vectors and Ang II Treatment

The siRNAs targeting Ndusf1 (si-Ndusf1) and the corresponding scrambled siRNAs were designed and synthesized by RiboBio (Guangzhou, China). The vector for Ndusf1 overexpression was constructed by cloning the full-length Ndusf1 sequence into the pcDNA3.1 vector, and the empty pcDNA3.1 vector was used as the corresponding negative control. All plasmids were purchased from RiboBio. For transfections, rat cardiomyocytes were seeded in 12-well plates and cultured for 24 h; then, cardiomyocytes were transfected with various plasmids or siRNAs by using Lipofectamine 2000 reagent (Invitrogen, Carlsbad, USA) according to the manufacturer's protocol. Cardiomyocytes were collected for the experiments 24 h after the transfection. For angiotensin II (Ang II; Sigma-Aldrich) treatment, cardiomyocytes were seeded in 12-well plates and cultured for 24 h; then, cardiomyocytes were treated with 100 nM Ang II for 24 h and harvested for subsequent experiments.

### 2.12. Quantitative Real-Time PCR (qRT-PCR)

Total RNA from cardiomyocytes and heart tissue was extracted using TRIzol reagent (Invitrogen, Carlsbad, USA) according to the manufacturer's protocol. RNA was reverse transcribed using a PrimerScript RT kit with gDNA eraser (Takara, Dalian, USA). Real-time PCR was performed using a SYBR Premix Ex Taq II kit (Takara) on an ABI7900 instrument (Applied Biosystems, Foster City, USA). The parameters for thermal cycling were as follows: 95°C for 15 s, 55°C for 15 s, and 72°C for 15 s for 40 cycles. The relative mRNA expression levels were determined by the comparative Ct method, and *β*-actin was used as the internal control.

### 2.13. Western Blot Assay

Proteins from cardiomyocytes or heart tissue were isolated using RIPA buffer supplemented with proteinase inhibitors (Sigma-Aldrich). The concentrations of the protein samples were measured by the BCA method. Equal amounts of proteins (50 *μ*g) were resolved by gel electrophoresis and transferred to a polyvinylidene difluoride (PVDF) membrane. After blocking with 5% nonfat milk at room temperature for 1 h, the membranes were incubated with primary antibodies against NDUFS1 (1 : 1,000; CST, Danvers, USA), atrial natriuretic peptide (ANP; 1 : 1,000; CST), brain natriuretic peptide (BNP; 1 : 1,000; CST), myosin heavy chain beta (*β*-MHC; 1 : 1,000; CST), and *β*-actin (1 : 2,000; CST) at 4°C overnight. Then, the membrane was incubated with horseradish peroxidase-conjugated secondary antibodies (1 : 2,000; CST) for 2 h at room temperature. The immunoreactive bands were analyzed by using a chemiluminescence system (Bio-Rad).

### 2.14. Assessment of mtDNA Copy Number

The mtDNA/nDNA ratio was evaluated by using the qRT-PCR assay as described previously. The primers were designed to target mtDNA (NADH dehydrogenase: 1,5′-AAACGCCCTAACAACCAT-3′ and 5′-GGATAGGATGC TCGGATT-3′) and nDNA (*β*-actin: 5′-ATGGTGGGAATGGGTCAGAA-3′ and 5′-CTTTTCACG GTTGGCCTTAG-3′). The relative mtDNA copy number was calculated by normalizing the mtDNA content to the expression of the *β*-actin gene.

### 2.15. Assessment of Mitochondrial Membrane Potential (MMP)

MMP of cardiomyocytes was evaluated using a JC-1 mitochondria staining kit (Thermo Fisher Scientific). Briefly, cardiomyocytes (5 × 10^3^ cells/well) were plated in 96-well plates, treated for 24, and incubated with JC-1 fluorescent dye for 20 min at room temperature in the dark. The fluorescent staining by JC-1 was evaluated by fluorescence microscopy. JC-1 monomers were imaged at excitation and emission wavelengths of 490 nm and 530 nm, respectively; JC-1 aggregates were imaged at excitation and emission wavelengths of 525 nm and 590 nm, respectively.

### 2.16. Detection of Mitochondrial ROS

The production of mitochondrial ROS was determined by using a MitoSOX fluorescent staining kit (Thermo Fisher Scientific, Waltham, USA) according to the manufacturer's protocol. Confocal laser scanning microscopy was used to capture fluorescent images, which were further analyzed using ImageJ software.

### 2.17. Flow Cytometry Analysis of ROS-Positive Cells

ROS production in cardiomyocytes was evaluated using the 2′,7′-dichlorofluorescein diacetate (DCF-DA) staining assay (Thermo Fisher Scientific). Briefly, the cells were incubated with DCF-DA for 30 min at 37°C in the dark, washed, resuspended in PBS, and maintained on ice for immediate assay by flow cytometry (BD Biosciences). The data were analyzed using FACSDiva software (BD) to calculate the number of ROS-positive cardiomyocytes.

### 2.18. Mitochondrial Mass Analysis Using MitoTracker Red Staining

MitoTracker Red staining was performed to assess mitochondrial mass. Briefly, cardiomyocytes were incubated with 100 nM MitoTracker Red for 30 min at 37°C. Fluorescence was detected at excitation and emission wavelengths of 490 and 516 nm, respectively, using an ELx-800 microplate reader (BioTek; Winooski, VT, USA).

### 2.19. Statistical Analysis

The data are presented as the mean ± standard deviation. All data analyses were performed using GraphPad Prism software (version 8; GraphPad Software, La Jolla, USA). Statistical significance of differences between various treatment groups was assessed using unpaired Student's *t*-test or one-way ANOVA followed by the Bonferroni multiple comparison test. *p* < 0.05 indicated statistical significance.

## 3. Results

### 3.1. scRNA-seq Clustering by the Seurat Package and Functional Enrichment Analysis

**Figure 1:**
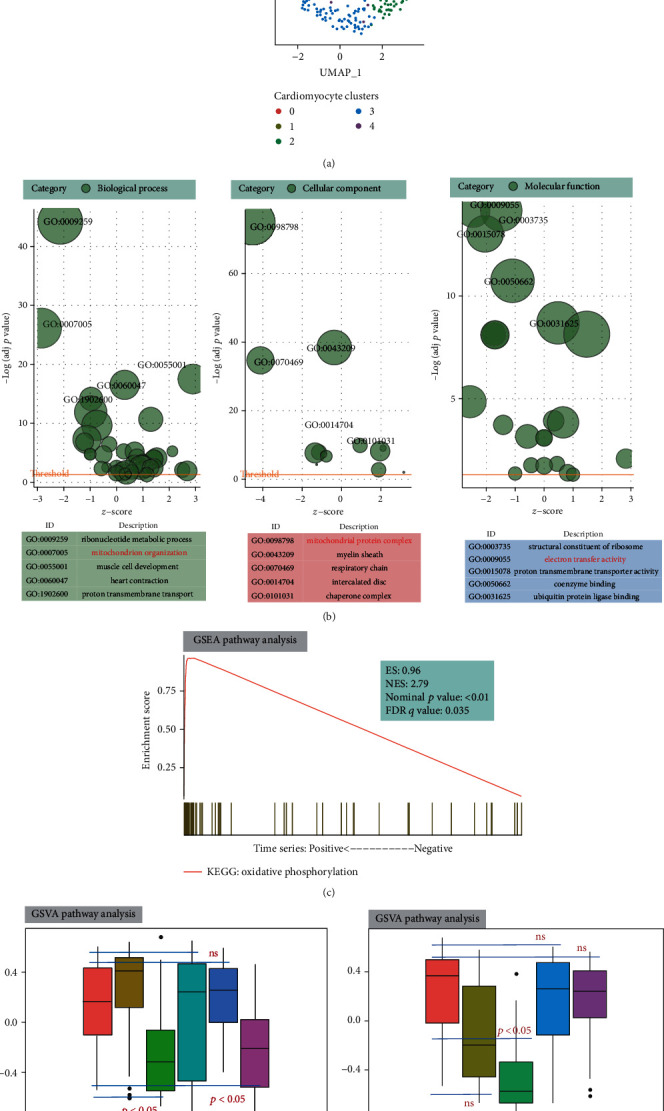
scRNA-seq clustering by the Seurat package and functional enrichment analysis. (a) UMAP visualization after batch correction. Cells are colored according to the clusters. (b) GO enrichment analysis of differentially expressed genes, including biological process, cellular component, and molecular function categories. (c) KEGG: oxidative phosphorylation pathway enrichment map of time series analysis during the development of a TAC model. (d) The patterns of the GSVA-calculated scores of the KEGG: oxidative phosphorylation pathway at various stages of cardiac hypertrophy and after sham operation. (e) The patterns of the GSVA-calculated scores of the KEGG: oxidative phosphorylation pathway across various cell clusters.

The number of principal components was set as 12, and cardiomyocytes were classified into six clusters based on UMAP visualization after batch correction (Figure 1(a)). A total of 3,408 highly variable genes were detected after normalization. Consequently, a total of 288 markers were identified by the Wilcoxon signed-rank test. These markers were then subjected to GO enrichment analysis. As shown in Figure 1(b) and Table S1, the genes were significantly enriched in “ribonucleotide metabolic process” (enriched genes = 97, *p* value = 1.44*E* − 48), “mitochondrion organization” (enriched genes = 77, *p* value = 3.35*E* − 29), “muscle cell development” (enriched genes = 43, *p* value = 1.72*E* − 20), “heart contraction” (enriched genes = 42, *p* value = 2.16*E* − 19), and “proton transmembrane transport” (enriched genes = 77, *p* value = 7.66*E* − 17) in the biological process category. Additionally, DE genes were significantly enriched in “mitochondrial protein complex” (enriched genes = 104, *p* value = 2.18*E* − 78), “myelin sheath” (enriched genes = 65, *p* value = 4.38*E* − 41), “respiratory chain” (enriched genes = 43, *p* value = 2.76*E* − 37), “intercalated disc” (enriched genes = 18, *p* value = 6.72*E* − 12), and “chaperone complex” (enriched genes = 11, *p* value = 4.69*E* − 11) in the cellular component category. For molecular function, the terms “structural constituent of ribosome” (enriched genes = 37, *p* value = 2.43*E* − 17), “electron transfer activity” (enriched genes = 24, *p* value = 2.73*E* − 17), “proton transmembrane transporter activity” (enriched genes = 29, *p* value = 5.49*E* − 16), “coenzyme binding” (enriched genes = 41, *p* value = 1.63*E* − 13), and “ubiquitin protein ligase binding” (enriched genes = 39, *p* value = 2.34*E* − 11) were also enriched.

As shown in Figure 1(c) and Table S2, GSEA was used to analyze the CH-related KEGG pathways, and the KEGG: oxidative phosphorylation pathway was significantly enriched in the progression of CH based on time series analysis (ES = 0.96, *S* = 2.79, nominal *p* value < 0.01, FDR *q* value = 0.035). Furthermore, the GSVA calculated scores of the KEGG: oxidative phosphorylation (OxP score) pathway at various stages of CH were distinctly different from those in the sham operation group. Specifically, CH-related scores were significantly lower in the TAC W1 and W4 groups compared to those in the sham group (all *p* value < 0.05; Figure 1(d) and Table S3). Moreover, the patterns of GSVA calculated based on the corresponding OxP scores across various cell clusters showed that the scores were significantly lower in cluster 2 than those in cluster 0 (*p* value < 0.05; Figure 1(e)).

### 3.2. Identification of Candidate Biomarkers Involved in TAC-Induced CH

**Figure 2:**
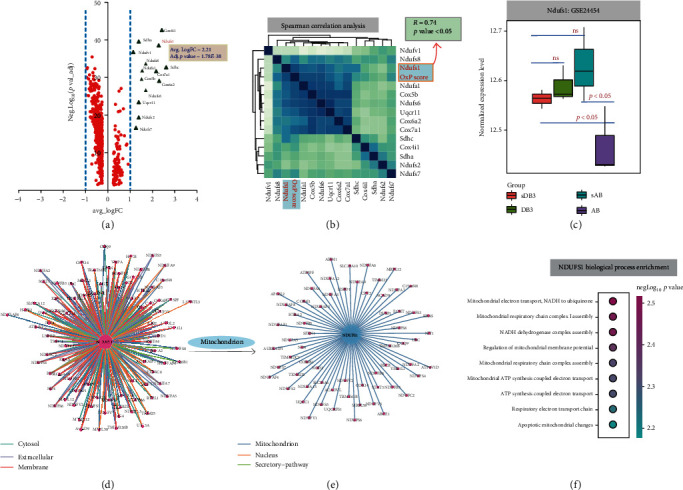
Identification of candidate biomarkers involved in TAC-induced CH. (a) Volcano plot of the differentially expressed genes in cluster 2 based on the *p* value and expression fold changes. (b) Spearman correlation analysis of genes from cluster 2 and GSVA scores of the KEGG: oxidative phosphorylation pathway in cluster 2. OxP score: GSVA scores of KEGG: oxidative phosphorylation. (c) The expression of Ndufs1 in mouse cardiac tissue based on the data of GSE24454. Mice were subjected to 4 weeks of aortic banding (AB) or sham procedure (sAB), banding and subsequent debanding (DB3), or sham procedure and subsequent debanding (sDB3) and sacrificed 3 days after debanding. (d) Protein-protein interaction network analysis of NDUFS1 based on the ComPPI database. (e) Protein-protein interaction network analysis of NDUFS1 in mitochondria. (f) Functional enrichment analysis of mitochondrial proteins interacting with NDUFS1.

DE genes in cluster 2 are illustrated by a volcano plot (Ndusf1: average LogFC = 2.21, adjusted *p* value = 1.78*E* − 38; Figure 2(a)). These genes included genes with log fold change > 1, which were used for Spearman correlation analysis; the results showed that Ndusf1 was highly correlated with the OxP score (*R* = 0.74, *p* value < 0.05; Figure 2(b)). The validation of Ndusf1 using GSE24454 showed that Ndusf1 was significantly downregulated in the heart tissue from aortic banding mice compared to that in sham-operated mice (Figure 2(c)). Additionally, NDUSF1 was identified as a key interactor in the protein-protein interaction network using ComPPI analysis and shared a close relationship with mitochondrial functional proteins, including STAT3, COX6B1, and ATP5PF (Figure 2(d), Figure 2(e), and Table S4). The biological process enrichment analysis showed that proteins that potentially interact with Ndusf1 were significantly associated with key mitochondrial functions, such as “mitochondrial electron transport, NADH to ubiquinone” (*p* value = 2.78*E* − 4), “mitochondrial respiratory chain complex I assembly” (*p* value = 1.52*E* − 3), and “NADH dehydrogenase complex assembly” (*p* value = 1.15*E* − 2) (Figure 2(f) and Table S5). Based on the results of bioinformatics analysis, Ndusf1 was selected for subsequent validation and functional analysis.

### 3.3. Ndufs1 Was Downregulated in the Hypertrophic Mouse Heart

**Figure 3:**
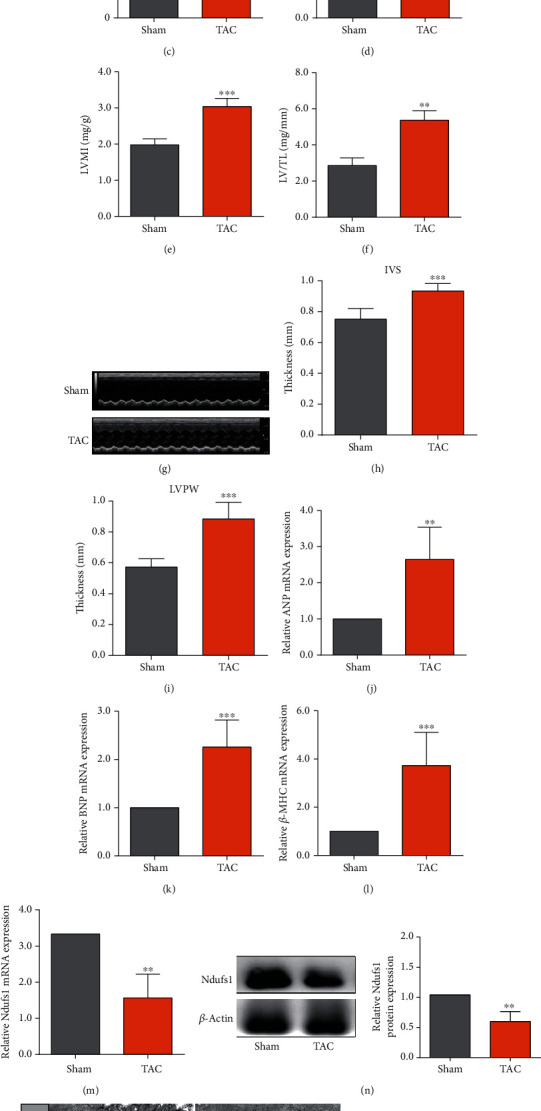
Ndufs1 was downregulated in the hypertrophic heart of mice. (a) H&E staining of mouse heart tissue in the sham and TAC groups. (b) The cardiomyocyte area was evaluated in the sham and TAC groups. The heart was dissected, and the heart weight (c), HMI (d), LVMI (e), and LV/TL (f) in the sham and TAC groups were determined. (g) Representative images of echocardiography in the sham and TAC groups, and the thickness of IVS (h) and LVPW (i) in the sham and TAC groups according to echocardiography. (j–l) The mRNA expression levels of ANP, BNP, and *β*-MHC in the heart tissue of the sham and TAC groups determined by qRT-PCR. (m, n) The mRNA and protein expression levels of Ndufs1 in the heart tissue of the sham and TAC groups were determined by qRT-PCR and western blot assays, respectively. (o) Representative TEM images of the heart tissue of the sham and TAC groups. (p, q) The mitochondrial volume and number were analyzed based on TEM images. *N* = 6. ^∗^*p* < 0.05, ^∗∗^*p* < 0.01, and ^∗∗∗^*p* < 0.001.

As shown in Figures 3(a) and 3(b), the results of H&E staining indicated that cardiomyocytes were significantly enlarged in the TAC group compared to those in the sham group (*p* < 0.05). Examination of the dissected heart tissue indicated that the heart weight, HMI, LVMI, and LV/TL were significantly higher in the TAC group than those in the sham group (Figures 3(c)–3(f); all *p* < 0.05). The results of echocardiography examination showed that the thickness of IVS and LVPW was significantly higher in the TAC group than that in the sham group (Figures 3(g)–3(i); all *p* < 0.05). qRT-PCR analysis showed that the mRNA expression levels of ANP, BNP, and *β*-MHC were significantly higher in heart tissue from the TAC group than those in the sham group (Figures 3(j)–3(l); all *p* < 0.05). Importantly, the expression levels of Ndusf1 mRNA and protein were significantly reduced in the heart tissue from the TAC group compared to those in the sham group (Figures 3(m) and 3(n); all *p* < 0.05). TEM examination showed that the mitochondrial volume was significantly increased, and the number of mitochondria was significantly decreased in the heart tissue from the TAC group compared to those in the control group (Figures 3(o)–3(q); all *p* < 0.05). The sarcomeres were enlarged, and myofibrils were disordered in the TAC group. Comparison with the sham group indicated that mitochondria in TAC-induced CH cardiomyocytes showed mild to moderate swelling, intact adventitia, edema of the matrix, disappearance of partial cristae, and aggregation between myofibrils.

### 3.4. Effects of Ang II on the Expression Levels of ANP, BNP, *β*-MHC, and Ndusf1

**Figure 4:**
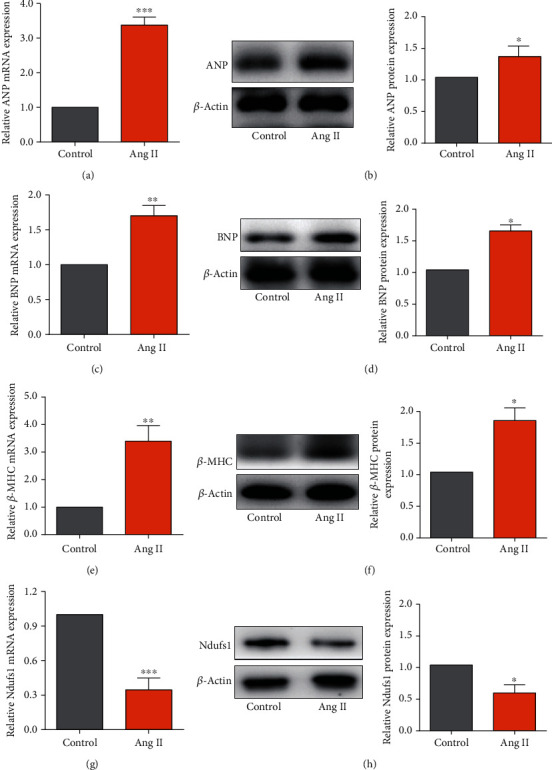
Ang II induced downregulation of Ndufs1 in rat cardiomyocytes. The mRNA and protein expression levels of ANP (a, b), BNP (c, d), *β*-MHC (e, f), and Ndufs1 (g, h) in control and Ang II-treated cardiomyocytes were determined by qRT-PCR and western blot assay. *N* = 3. ^∗^*p* < 0.05, ^∗∗^*p* < 0.01, and ^∗∗∗^*p* < 0.001.

As shown in Figure 4, Ang II treatment caused a significant increase in the mRNA and protein expression levels of ANP, BNP, and *β*-MHC in cardiomyocytes compared to those in the control group (Figures 4(a)–4(f); all *p* < 0.05). On the other hand, the mRNA and protein expression levels of Ndusf1 in cardiomyocytes were significantly reduced by Ang II treatment compared to those in the control group (Figures 4(g) and 4(h); all *p* < 0.05). These results indicated that Ndusf1 may have certain biological functions in Ang II-stimulated cardiomyocytes.

### 3.5. Effects of Ndusf1 Knockdown on the Expression Levels of ANP, BNP, and *β*-MHC and on Mitochondrial Functions

**Figure 5:**
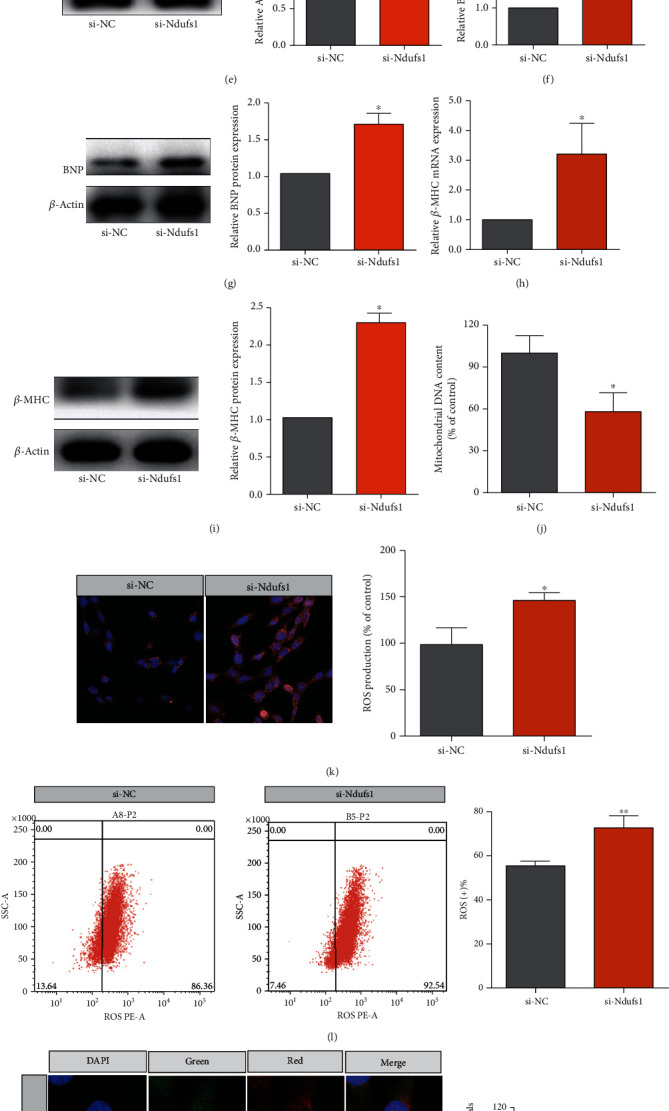
Effects of Ndufs1 knockdown on the expression of hypertrophic markers and mitochondrial function. (a, b) The mRNA and protein expression levels of Ndufs1 in cardiomyocytes transfected with scrambled siRNA or Ndufs1 siRNA were determined by qRT-PCR and western blot assays. (c) The relative cardiomyocyte area was determined in cardiomyocytes transfected with scrambled siRNA or Ndufs1 siRNA. The mRNA and protein expression levels of ANP (d, e), BNP (f, g), and *β*-MHC (h, i) in cardiomyocytes transfected with scrambled siRNA or Ndufs1 siRNA were determined by qRT-PCR and western blot assays. The mitochondrial DNA content (j), mitochondrial ROS production (k), ROS-positive cardiomyocytes (l), MMP (m), and mitochondrial mass (n) were evaluated in cardiomyocytes transfected with scrambled siRNA or Ndufs1 siRNA. *N* = 3. ^∗^*p* < 0.05, ^∗∗^*p* < 0.01, and ^∗∗∗^*p* < 0.001.

Ndusf1 was silenced by transient transfection of cardiomyocytes with Ndusf1 siRNA. As shown in Figures 5(a) and 5(b), Ndusf1 siRNA transfection (si-Ndusf1) significantly reduced the expression levels of Ndusf1 mRNA and protein in cardiomyocytes compared to that in cells transfected with scrambled siRNA (si-NC; all *p* < 0.05). The size of cardiomyocytes was significantly increased in the si-Ndufs1 group compared with that in the si-NC group (Figure 5(c); *p* < 0.01). The results of qRT-PCR and western blot indicated that the expression levels of mRNA and protein of ANP, BNP, and *β*-MHC were significantly downregulated in si-Ndusf1-transfected cardiomyocytes compared to those in si-NC-transfected cardiomyocytes (Figures 5(d)–5(i); all *p* < 0.05). Furthermore, the effects of Nudsf1 knockdown on mitochondrial functions were determined by measuring mitochondrial DNA content, ROS production, and MMP. As shown in Figure 5(j), Ndusf1 knockdown significantly reduced the mitochondrial DNA content in cardiomyocytes compared to that in the si-NC group (*p* < 0.01). Consistently, the levels of ROS production in cardiomyocytes were significantly elevated after Ndusf1 silencing (Figures 5(k) and 5(l); all *p* < 0.05). Moreover, the fluorescent staining results showed that Ndufs1 knockdown dramatically increased MMP and mitochondrial mass of cardiomyocytes compared to those in the si-NC group (Figures 5(m) and 5(n); all *p* < 0.05). These results indicate that silencing Ndusf1 gene expression may aggravate MMP damage in Ang II-induced myocardial hypertrophy.

### 3.6. Effects of Ndusf1 Overexpression on Ang II-Induced Hypertrophy of Cardiomyocytes

**Figure 6:**
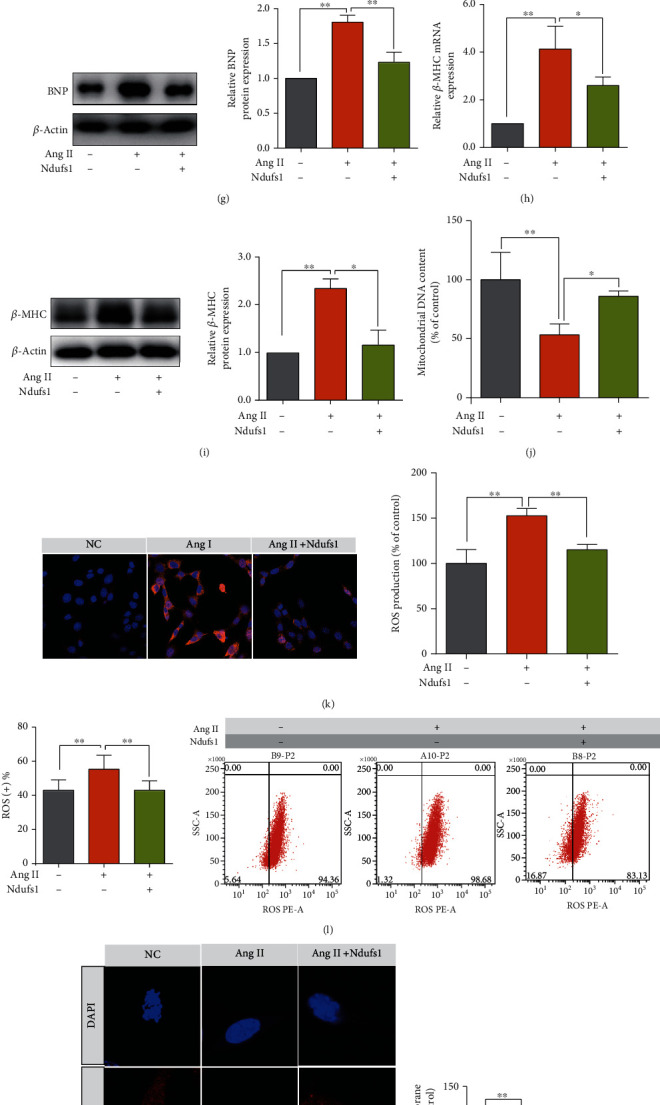
Ndufs1 overexpression attenuated Ang II-induced cardiac hypertrophy and mitochondrial dysfunction in cardiomyocytes. (a, b) The mRNA and protein expression levels of Ndufs1 in cardiomyocytes transfected with pcDNA3.1 or pcDNA3.1-Ndufs1 were determined by qRT-PCR and western blot assays. (c) The relative cardiomyocyte area was determined in cardiomyocytes subjected to various treatments. The mRNA and protein expression levels of ANP (d, e), BNP (f, g), and *β*-MHC (h, i) in cardiomyocytes subjected to various treatments were determined by qRT-PCR and western blot assays. The mitochondrial DNA content (j), mitochondrial ROS production (k), ROS-positive cardiomyocytes (l), MMP (m), and mitochondrial mass (n) were evaluated in cardiomyocytes subjected to various treatments. *N* = 3. ^∗^*p* < 0.05, ^∗∗^*p* < 0.01, and ^∗∗∗^*p* < 0.001.

Initially, we constructed the vector for Ndusf1 overexpression using the pcDNA3.1 plasmids. Cardiomyocytes transfected with the vector for Ndusf1 overexpression exhibited a significant increase in the levels of Ndusf1 mRNA and protein compared with those in the cells transfected with pcDNA3.1 (NC group; Figures 6(a) and 6(b); all *p* < 0.05). As expected, Ang II treatment increased the cardiomyocyte area compared with that in the control group, and Ndusf1 overexpression attenuated Ang II-induced increase in the cardiomyocyte area (Figure 6(c); *p* < 0.05). Consistently, Ang II treatment caused a significant increase in the levels of mRNA and protein of ANP, BNP, and *β*-MHC (all *p* < 0.05), and Ndusf1 overexpression partially counteracted the stimulatory effects of Ang II on ANP, BNP, and *β*-MHC expression in cardiomyocytes (Figures 6(d)–6(i); all *p* < 0.05). The mitochondrial DNA content of cardiomyocytes was significantly reduced by Ang II, and this effect was attenuated by Ndusf1 overexpression (Figure 6(j); all *p* < 0.05). Moreover, Ndusf1 overexpression attenuated Ang II-induced elevation in the ROS production levels in cardiomyocytes (Figures 6(k) and 6(l); all *p* < 0.05). Importantly, Ang II treatment significantly repressed MMP and mitochondrial mass in cardiomyocytes, and the effect was significantly alleviated by overexpression of Ndusf1 (Figures 6(m) and 6(n)). In turn, these results indicate that Ndusf1 overexpression may protect against MMP disorder in Ang II-induced mitochondrial dysfunction.

## 4. Discussion

CH is a pathophysiological response characterized by increased thickness of the ventricular wall, greater myocardial cell volume, and enhanced myocardial contractility in the early stage of overload pressure [1, 3]. The pathophysiology of CH is complex and involves multiple cellular events, and the mechanisms of the development of CH are not fully understood [1, 3, 30]. Growing evidence indicates that mitochondrial dysfunction is closely related to the development and progression of CH. In the present study, we explored the GSE95140 datasets by using integrated bioinformatics analysis and demonstrated downregulation of Ndusf1 in heart tissue of mice with CH. The expression of Ndusf1 was further validated in a mouse TAC model; our data showed that Ndusf1 expression was significantly downregulated in hypertrophic heart tissue compared to that in normal controls. Moreover, *in vitro* mechanistic studies showed that Ndusf1 knockdown induced cardiomyocyte hypertrophy, decreased mitochondrial DNA content and MMP, and increased mitochondrial ROS production in cardiomyocytes. On the other hand, Ang II treatment upregulated the expression levels of ANP, BNP, and *β*-MHC, decreased mitochondrial DNA content and MMP, and increased mitochondrial ROS production in cardiomyocytes; moreover, Ang II-mediated effects were significantly attenuated by overexpression of Ndusf1 in rat cardiomyocytes. Overall, our results indicate the important roles of Ndusf1 in the development and progression of CH.

In the present study, TAC surgery was performed to establish a CH mouse model. Enlarged cardiomyocytes and increases in the heart weight, HMI, LVMI, LV/TL, and thickness of IVS and LVPW were detected in mice subjected to TAC compared with those in sham-operated mice; these findings are consistent with the results of previous studies [31]. Additionally, the mRNA expression levels of ANP, BNP, and *β*-MHC were elevated in the heart tissue of the mice with CH. ANP, BNP, and *β*-MHC are commonly used cardiac hypertrophic biomarkers, and elevated levels of these biomarkers have been demonstrated in heart tissue in clinical studies and animal models [32–35]. Overall, these results suggest successful establishment of CH in mice subjected to TAC treatment. Further validation by qRT-PCR and western blot showed that the expression levels of Ndusf1 mRNA and protein were downregulated in hypertrophic heart tissue. The data suggest that Ndusf1 may play an important role in CH.

Mitochondria are considered to be dynamic “energy stations”, and these morphological changes are characterized by fragmented disconnection and elongated interconnection of mitochondria, which are regulated by activation of mitochondrial fission and fusion proteases or posttranslational modifications of proteins during the development of CH [1, 36]. Ndusf1 (NADH:ubiquinone oxidoreductase core subunit S1) is the largest subunit of complex I; the corresponding gene encodes a 75 kDa subunit of NADH-ubiquinone oxidoreductase [37] and thus has gained particular attention due to its significant role in the activity of mitochondrial oxidative phosphorylation. Mutations or deficiency of this gene can destabilize complex I assembly and result in defects of the activity of the electron transport chain (ETC) and elevated ATP production, which can cause mitochondrial fusion and fission to restore damaged mitochondrial DNA and remove damaged mitochondrial fragments, respectively [38–40]. However, the role of Ndusf1 in the pathophysiology of CH has not been reported; however, the importance of Ndusf1 in cardiovascular diseases has been emphasized by several research groups. Qi et al. showed that Akap1 deficiency exacerbates diabetic cardiomyopathy in mice by Ndusf1-mediated mitochondrial dysfunction and apoptosis [19]. Ndusf1 was shown to be upregulated in cardiac cells upon cyclic stretch, which may be associated with mitochondrial biogenesis [41]. Sato et al. showed that Ndusf1 is associated with the cardiac response to iron deficiency [42]. Additionally, the cardiac levels of acetylated forms of Ndusf1 are decreased in caloric restriction [43]. Impaired electron transport chains in mitochondria contribute to the development and progression of CH [44, 45]. Ndusf1 is one of the key regulators of the electron transport chain; thus, we speculate that Ndusf1 may be a key factor in CH. The results of our in vitro studies indicated that the Ang II-induced increase of the expression of hypertrophic biomarkers was accompanied by downregulation of Ndusf1 in cardiomyocytes. Ang II is widely used to induce a cellular model of CH in cardiomyocytes [46, 47]. Schwartz et al. used serial analysis of gene expression to demonstrate that continuous Ang II treatment induces downregulation of Ndusf1 in the mouse heart [48]. The activation of the endogenous renin-angiotensin aldosterone system increases the protein levels of Ndusf1 in urine in hypertensive patients [49]. Additionally, Ang II exacerbates mitochondrial dysfunction and oxidative stress to cause heart failure [50]. Thus, in combination with these findings, our results may imply that Ang II-induced CH in cardiomyocytes may be associated with downregulation of Ndusf1.

To gain additional insight into the mechanism of action of Ndusf1 in Ang II-induced CH, we performed loss- and gain-of-function studies in cardiomyocytes. Our results showed that Ndusf1 knockdown decreased the mitochondrial DNA content and MMP and increased mitochondrial ROS production; in addition, Ndusf1 knockdown increased the expression levels of hypertrophic biomarkers in cardiomyocytes, possibly indicating that Ndusf1 knockdown promoted CH by impairing mitochondrial functions. Downregulation of Ndusf1 inhibits the neuroprotective effects of pyrroloquinoline against rotenone injury in cultured SH-SY5Y cells and cultured midbrain neurons, and rotenone can impair mitochondrial dysfunction [51]. Lopez-Fabuel et al. showed that Ndusf1 knockdown impairs mitochondrial O_2_ consumption and increases ROS production in neurons [52]. On the other hand, our results showed that overexpression of Ndusf1 attenuated Ang II-mediated effects in cardiomyocytes. Thus, Ndusf1 may be involved in Ang II-mediated CH by modulating mitochondrial functions in cardiomyocytes.

Balanced regulation of mitochondrial fusion and fission is very delicate and susceptible to the development of CH and heart failure; thus, efficient myocardial therapies that target mitochondrial lesions and dysfunction may be a new strategy to suppress susceptibility to myocardial remodeling and attenuate myocardial fibrosis; however, these possibilities are poorly characterized [1, 5, 11]. Ndufs1 has been illustrated to play a central role in the regulation of morphological dynamics and oxidative stress and may play an important role in mitochondrial crista remodeling, cytochrome release, and mitochondrial respiration in the development of CH and heart failure. A decrease in the expression of Ndufs1, in turn, contributes to aggravated mitochondrial membrane potential disorder, mitochondrial ROS production, and mitochondrial DNA damage.

In conclusion, our results demonstrated downregulation of Ndusf1 in hypertrophic heart tissue, and the results of the mechanistic studies suggest that Ndusf1 deficiency causes mitochondrial dysfunction in cardiomyocytes, which may be associated with the development and progression of CH.

## 5. Limitations

There are several limitations in the present study. First, the expression of Ndusf1 in the mouse cardiac tissue was determined 4 weeks after TAC surgery in mice, which cannot reflect the changes in Ndusf1 during the progression of CH. Thus, future studies may determine the expression of Ndusf1 at various time points to confirm the role of Ndusf1 in the progression of CH. Second, the expression of Ndusf1 was validated only in mouse cardiac tissue and cardiomyocytes, and future studies should determine the expression of Ndusf1 in the clinical samples of hypertrophic hearts. Third, loss- and gain-of-function studies were only performed in vitro, and the functional role of Ndusf1 in vivo in the pathophysiology of CH should be determined. Fourth, the downstream signaling pathways mediated by Ndusf1 should be further explored.

## Data Availability

Both of the GSE95140 and GSE24454 datasets were obtained from the GEO (https://www.ncbi.nlm.nih.gov/geo/) database, and the analysis scripts would be accessed from Dr. Rongjun Zou on a request.

## Supplementary Materials

Supplementary Materials Additional file 1 Table S1: Gene Ontology enrichment terms for differentially expressed genes identified based on the scRNA-seq of pressure overload-induced myocardial hypertrophy. Additional file 2: Table S2: pathways correlated with pressure overload-induced myocardial hypertrophy based on time series-related GSEA. Additional file 3: Table S3: the oxidative phosphorylation score of single cardiomyocytes was calculated by GSVA. Additional file 4: Table S4: the cellular compartment-specific protein-protein interaction network of NDUFS1. Additional file 5: Table S5: biological process enrichment analyses of NDUFS1.

## References

[1] Nakamura M., Sadoshima J. (2018). Mechanisms of physiological and pathological cardiac hypertrophy. *Nature Reviews Cardiology*.

[2] Samak M., Fatullayev J., Sabashnikov A. (2016). Cardiac hypertrophy: an introduction to molecular and cellular basis. *Medical Science Monitor Basic Research*.

[3] Shimizu I., Minamino T. (2016). Physiological and pathological cardiac hypertrophy. *Journal of Molecular and Cellular Cardiology*.

[4] Tham Y. K., Bernardo B. C., Ooi J. Y., Weeks K. L., McMullen J. R. (2015). Pathophysiology of cardiac hypertrophy and heart failure: signaling pathways and novel therapeutic targets. *Archives of Toxicology*.

[5] Oldfield C. J., Duhamel T. A., Dhalla N. S. (2020). Mechanisms for the transition from physiological to pathological cardiac hypertrophy. *Canadian Journal of Physiology and Pharmacology*.

[6] Facundo H. D., Brainard R. E., de Lemos Caldas F. R., Lucas A. M. (2017). Mitochondria and cardiac hypertrophy. *Advances in Experimental Medicine and Biology*.

[7] Rosca M. G., Tandler B., Hoppel C. L. (2013). Mitochondria in cardiac hypertrophy and heart failure. *Journal of Molecular and Cellular Cardiology*.

[8] Heusch G. (2019). Coronary microvascular obstruction: the new frontier in cardioprotection. *Basic Research in Cardiology*.

[9] Tsutsui H., Kinugawa S., Matsushima S. (2011). Oxidative stress and heart failure. *American Journal of Physiology. Heart and Circulatory Physiology*.

[10] Zhou B., Tian R. (2018). Mitochondrial dysfunction in pathophysiology of heart failure. *The Journal of Clinical Investigation*.

[11] Hughes W. E., Beyer A. M., Gutterman D. D. (2020). Vascular autophagy in health and disease. *Basic Research in Cardiology*.

[12] Pham T., Loiselle D., Power A., Hickey A. J. (2014). Mitochondrial inefficiencies and anoxic ATP hydrolysis capacities in diabetic rat heart. *American Journal of Physiology. Cell Physiology*.

[13] Power A., Pearson N., Pham T., Cheung C., Phillips A., Hickey A. (2014). Uncoupling of oxidative phosphorylation and ATP synthase reversal within the hyperthermic heart. *Physiological Reports*.

[14] Marín-García J., Akhmedov A. T., Moe G. W. (2013). Mitochondria in heart failure: the emerging role of mitochondrial dynamics. *Heart Failure Reviews*.

[15] Garza-Cervantes J. A., Ramos-González M., Lozano O., Jerjes-Sánchez C., García-Rivas G. (2020). Therapeutic applications of cannabinoids in cardiomyopathy and heart failure. *Oxidative Medicine and Cellular Longevity*.

[16] Martín-Fernández B., Gredilla R. (2016). Mitochondria and oxidative stress in heart aging. *Age (Dordrecht, Netherlands)*.

[17] Capasso T. L., Li B., Volek H. J. (2020). BMP10-mediated ALK1 signaling is continuously required for vascular development and maintenance. *Angiogenesis*.

[18] Ni Y., Hagras M. A., Konstantopoulou V., Mayr J. A., Stuchebrukhov A. A., Meierhofer D. (2019). Mutations in NDUFS1 cause metabolic reprogramming and disruption of the electron transfer. *Cell*.

[19] Qi B., He L., Zhao Y. (2020). Akap1 deficiency exacerbates diabetic cardiomyopathy in mice by NDUFS1-mediated mitochondrial dysfunction and apoptosis. *Diabetologia*.

[20] Zhu Y., Wang Z., Ni J. (2015). Genetic variant in NDUFS1 gene is associated with schizophrenia and negative symptoms in Han Chinese. *Journal of Human Genetics*.

[21] Nomura S., Satoh M., Fujita T. (2018). Cardiomyocyte gene programs encoding morphological and functional signatures in cardiac hypertrophy and failure. *Nature Communications*.

[22] McCarthy D. J., Campbell K. R., Lun A. T., Wills Q. F. (2017). Scater: pre-processing, quality control, normalization and visualization of single-cell RNA-seq data in R. *Bioinformatics*.

[23] Butler A., Hoffman P., Smibert P., Papalexi E., Satija R. (2018). Integrating single-cell transcriptomic data across different conditions, technologies, and species. *Nature Biotechnology*.

[24] Bjørnstad J. L., Sjaastad I., Nygård S. (2011). Collagen isoform shift during the early phase of reverse left ventricular remodelling after relief of pressure overload. *European Heart Journal*.

[25] Yu G., Wang L. G., Han Y., He Q. Y. (2012). clusterProfiler: an R package for comparing biological themes among gene clusters. *OMICS*.

[26] Chen J., Bardes E. E., Aronow B. J., Jegga A. G. (2009). ToppGene Suite for gene list enrichment analysis and candidate gene prioritization. *Nucleic Acids Research*.

[27] Veres D. V., Gyurkó D. M., Thaler B. (2015). ComPPI: a cellular compartment-specific database for protein-protein interaction network analysis. *Nucleic Acids Research*.

[28] Subramanian A., Tamayo P., Mootha V. K. (2005). Gene set enrichment analysis: a knowledge-based approach for interpreting genome-wide expression profiles. *Proceedings of the National Academy of Sciences of the United States of America*.

[29] Hänzelmann S., Castelo R., Guinney J. (2013). GSVA: gene set variation analysis for microarray and RNA-seq data. *BMC Bioinformatics*.

[30] di Somma M., Vliora M., Grillo E. (2020). Role of VEGFs in metabolic disorders. *Angiogenesis*.

[31] Mishra S., Ling H., Grimm M., Zhang T., Bers D. M., Brown J. H. (2010). Cardiac hypertrophy and heart failure development through Gq and CaM kinase II signaling. *Journal of Cardiovascular Pharmacology*.

[32] Fulgencio-Covián A., Alonso-Barroso E., Guenzel A. J. (2020). Pathogenic implications of dysregulated miRNAs in propionic acidemia related cardiomyopathy. *Translational Research*.

[33] Kar D., Bandyopadhyay A. (2018). Targeting peroxisome proliferator activated receptor *α* (PPAR *α*) for the prevention of mitochondrial impairment and hypertrophy in cardiomyocytes. *Cellular Physiology and Biochemistry*.

[34] Yim J., Cho H., Rabkin S. W. (2018). Gene expression and gene associations during the development of heart failure with preserved ejection fraction in the Dahl salt sensitive model of hypertension. *Clinical and Experimental Hypertension*.

[35] Zhang C., Wang Y., Ge Z. (2018). GDF11 attenuated ANG II-induced hypertrophic cardiomyopathy and expression of ANP, BNP and beta-MHC through down-regulating CCL11 in mice. *Current Molecular Medicine*.

[36] Wang J., Zhu P., Li R., Ren J., Zhou H. (2020). Fundc1-dependent mitophagy is obligatory to ischemic preconditioning-conferred renoprotection in ischemic AKI via suppression of Drp1-mediated mitochondrial fission. *Redox Biology*.

[37] Iuso A., Scacco S., Piccoli C. (2006). Dysfunctions of cellular oxidative metabolism in patients with mutations in the NDUFS1 and NDUFS4 genes of complex I. *The Journal of Biological Chemistry*.

[38] Hirst J. (2013). Mitochondrial complex I. *Annual Review of Biochemistry*.

[39] Jusic A., Devaux Y. (2020). Mitochondrial noncoding RNA-regulatory network in cardiovascular disease. *Basic Research in Cardiology*.

[40] Signorello M. G., Ravera S., Leoncini G. (2020). Lectin-induced oxidative stress in human platelets. *Redox Biology*.

[41] Kim H. K., Kang Y. G., Jeong S. H. (2018). Cyclic stretch increases mitochondrial biogenesis in a cardiac cell line. *Biochemical and Biophysical Research Communications*.

[42] Sato T., Chang H. C., Bayeva M. (2018). mRNA-binding protein tristetraprolin is essential for cardiac response to iron deficiency by regulating mitochondrial function. *Proceedings of the National Academy of Sciences of the United States of America*.

[43] Shinmura K., Tamaki K., Sano M. (2011). Caloric restriction primes mitochondria for ischemic stress by deacetylating specific mitochondrial proteins of the electron transport chain. *Circulation Research*.

[44] Griffiths E. R., Friehs I., Scherr E., Poutias D., McGowan F. X., Del Nido P. J. (2010). Electron transport chain dysfunction in neonatal pressure-overload hypertrophy precedes cardiomyocyte apoptosis independent of oxidative stress. *The Journal of Thoracic and Cardiovascular Surgery*.

[45] Osterholt M., Nguyen T. D., Schwarzer M., Doenst T. (2013). Alterations in mitochondrial function in cardiac hypertrophy and heart failure. *Heart Failure Reviews*.

[46] Luo Y. X., Tang X., An X. Z. (2017). SIRT4 accelerates Ang II-induced pathological cardiac hypertrophy by inhibiting manganese superoxide dismutase activity. *European Heart Journal*.

[47] Wang L., Zhang Y. L., Lin Q. Y. (2018). CXCL1-CXCR2 axis mediates angiotensin II-induced cardiac hypertrophy and remodelling through regulation of monocyte infiltration. *European Heart Journal*.

[48] Schwartz F., Duka A., Duka I., Cui J., Gavras H. (2004). Novel targets of ANG II regulation in mouse heart identified by serial analysis of gene expression. *American Journal of Physiology-Heart and Circulatory Physiology*.

[49] Qi Y., Wang X., Rose K. L. (2016). Activation of the endogenous renin-angiotensin-aldosterone system or aldosterone administration increases urinary exosomal sodium channel excretion. *Journal of the American Society of Nephrology*.

[50] Hamilton D. J., Zhang A., Li S. (2016). Combination of angiotensin II and l-NG-nitroarginine methyl ester exacerbates mitochondrial dysfunction and oxidative stress to cause heart failure. *American Journal of Physiology-Heart and Circulatory Physiology*.

[51] Zhang Q., Chen S., Yu S. (2016). Neuroprotective effects of pyrroloquinoline quinone against rotenone injury in primary cultured midbrain neurons and in a rat model of Parkinson’s disease. *Neuropharmacology*.

[52] Lopez-Fabuel I., Le Douce J., Logan A. (2016). Complex I assembly into supercomplexes determines differential mitochondrial ROS production in neurons and astrocytes. *Proceedings of the National Academy of Sciences of the United States of America*.

