# Supplementary material for: Ndufs1 Deficiency Aggravates the Mitochondrial Membrane Potential Dysfunction in Pressure Overload-Induced Myocardial Hypertrophy
Source: Oxid Med Cell Longev. 2021 Mar 3;2021:5545261. doi: 10.1155/2021/5545261 (PMC7952157; doi:10.1155/2021/5545261)
Supplement: Supplementary Materials — Additional file 1 Table S1: Gene Ontology enrichment terms for differentially expressed genes identified based on the scRNA-seq of pressure overload-induced myocardial hypertrophy. Additional file 2: Table S2: pathways correlated with pressure overload-induced myocardial hypertrophy based on time series-related GSEA. Additional file 3: Table S3: the oxidative phosphorylation score of single cardiomyocytes was calculated by GSVA. Additional file 4: Table S4: the cellular compartment-specific protein-protein interaction network of NDUFS1. Additional file 5: Table S5: biological process enrichment analyses of NDUFS1. [file 5545261.f1.zip › Table S1 Gene ontology term enrichment analysis.doc]

**Table S1 Gene ontology term enrichment analysis**

| **GO term: biological processes** | | | | |  |
| --- | --- | --- | --- | --- | --- |
|  |  |  |  |  |  |
| ID | Description | GeneRatio | pvalue | p.adjust |  |
| GO:0009259 | ribonucleotide metabolic process | 97/861 | 1.44E-48 | 6.72E-45 |  |
| GO:0007005 | mitochondrion organization | 77/861 | 3.35E-29 | 7.10E-27 |  |
| GO:0055001 | muscle cell development | 43/861 | 1.72E-20 | 3.08E-18 |  |
| GO:0060047 | heart contraction | 42/861 | 2.16E-19 | 2.97E-17 |  |
| GO:1902600 | proton transmembrane transport | 27/861 | 7.66E-17 | 7.15E-15 |  |
| GO:0006979 | response to oxidative stress | 51/861 | 1.68E-14 | 1.22E-12 |  |
| GO:0006457 | protein folding | 29/861 | 3.38E-13 | 2.10E-11 |  |
| GO:0072594 | establishment of protein localization to organelle | 45/861 | 4.24E-12 | 2.22E-10 |  |
| GO:0062012 | regulation of small molecule metabolic process | 39/861 | 1.42E-09 | 5.38E-08 |  |
| GO:0051881 | regulation of mitochondrial membrane potential | 17/861 | 3.85E-09 | 1.40E-07 |  |
| GO:1903115 | regulation of actin filament-based movement | 13/861 | 1.02E-08 | 3.54E-07 |  |
| GO:0035994 | response to muscle stretch | 8/861 | 1.85E-07 | 5.45E-06 |  |
| GO:0043462 | regulation of ATPase activity | 13/861 | 1.95E-07 | 5.69E-06 |  |
| GO:0055076 | transition metal ion homeostasis | 19/861 | 2.09E-07 | 6.04E-06 |  |
| GO:0099131 | ATP hydrolysis coupled ion transmembrane transport | 9/861 | 6.63E-07 | 1.69E-05 |  |
| GO:0099132 | ATP hydrolysis coupled cation transmembrane transport | 9/861 | 6.63E-07 | 1.69E-05 |  |
| GO:0062014 | negative regulation of small molecule metabolic process | 16/861 | 7.25E-07 | 1.84E-05 |  |
| GO:0120162 | positive regulation of cold-induced thermogenesis | 15/861 | 2.48E-06 | 5.61E-05 |  |
| GO:0001659 | temperature homeostasis | 21/861 | 3.38E-06 | 7.44E-05 |  |
| GO:0106106 | cold-induced thermogenesis | 18/861 | 5.63E-06 | 0.000116 |  |
| GO:0120161 | regulation of cold-induced thermogenesis | 18/861 | 5.63E-06 | 0.000116 |  |
| GO:1905710 | positive regulation of membrane permeability | 9/861 | 7.99E-06 | 0.000158 |  |
| GO:1905475 | regulation of protein localization to membrane | 19/861 | 6.82E-05 | 0.001011 |  |
| GO:0051604 | protein maturation | 25/861 | 0.000163 | 0.002123 |  |
| GO:0044794 | positive regulation by host of viral process | 5/861 | 0.000217 | 0.002671 |  |
| GO:0061919 | process utilizing autophagic mechanism | 29/861 | 0.000251 | 0.003017 |  |
| GO:1905037 | autophagosome organization | 12/861 | 0.000252 | 0.003017 |  |
| GO:0014823 | response to activity | 7/861 | 0.000258 | 0.003069 |  |
| GO:0099173 | postsynapse organization | 18/861 | 0.000289 | 0.003383 |  |
| GO:0010634 | positive regulation of epithelial cell migration | 15/861 | 0.000289 | 0.003383 |  |
| GO:0140053 | mitochondrial gene expression | 12/861 | 0.000441 | 0.004652 |  |
| GO:0010811 | positive regulation of cell-substrate adhesion | 13/861 | 0.000839 | 0.007884 |  |
| GO:0110053 | regulation of actin filament organization | 20/861 | 0.001363 | 0.011792 |  |
| GO:1905477 | positive regulation of protein localization to membrane | 11/861 | 0.001435 | 0.012191 |  |
| GO:0043393 | regulation of protein binding | 19/861 | 0.00175 | 0.014439 |  |
| GO:0097006 | regulation of plasma lipoprotein particle levels | 8/861 | 0.001812 | 0.014874 |  |
| GO:0055089 | fatty acid homeostasis | 4/861 | 0.001861 | 0.015036 |  |
| GO:0099175 | regulation of postsynapse organization | 12/861 | 0.002266 | 0.017672 |  |
| GO:0106057 | negative regulation of calcineurin-mediated signaling | 4/861 | 0.002408 | 0.018532 |  |
| GO:0071900 | regulation of protein serine/threonine kinase activity | 28/861 | 0.002622 | 0.019723 |  |
| GO:0042594 | response to starvation | 14/861 | 0.003689 | 0.026525 |  |
| GO:0045773 | positive regulation of axon extension | 7/861 | 0.003692 | 0.026525 |  |
| GO:1905954 | positive regulation of lipid localization | 9/861 | 0.005795 | 0.038112 |  |
| GO:0018208 | peptidyl-proline modification | 5/861 | 0.006999 | 0.044344 |  |
| GO:1905952 | regulation of lipid localization | 12/861 | 0.007973 | 0.049507 |  |
| GO term:cellular components | | | | |  |
|  |  |  |  |  |  |
| ID | Description | GeneRatio | pvalue | p.adjust |  |
| GO:0098798 | mitochondrial protein complex | 104/875 | 2.18E-78 | 1.27E-75 |  |
| GO:0043209 | myelin sheath | 65/875 | 4.38E-41 | 3.65E-39 |  |
| GO:0070469 | respiratory chain | 43/875 | 2.76E-37 | 2.01E-35 |  |
| GO:0014704 | intercalated disc | 18/875 | 6.72E-12 | 1.31E-10 |  |
| GO:0101031 | chaperone complex | 11/875 | 4.69E-11 | 8.54E-10 |  |
| GO:0042383 | sarcolemma | 26/875 | 4.10E-10 | 6.13E-09 |  |
| GO:1905368 | peptidase complex | 19/875 | 8.97E-10 | 1.28E-08 |  |
| GO:0019867 | outer membrane | 27/875 | 1.64E-09 | 2.03E-08 |  |
| GO:0070069 | cytochrome complex | 11/875 | 5.33E-09 | 5.86E-08 |  |
| GO:1905369 | endopeptidase complex | 15/875 | 1.94E-08 | 1.83E-07 |  |
| GO:0009295 | nucleoid | 10/875 | 7.46E-06 | 4.94E-05 |  |
| GO:0043197 | dendritic spine | 18/875 | 0.000331 | 0.001709 |  |
| GO:0070062 | extracellular exosome | 10/875 | 0.001944 | 0.008995 |  |
| GO term: molecular functions | | | | |  |
|  |  |  |  |  |  |
| ID | Description | GeneRatio | pvalue | p.adjust |  |
| GO:0003735 | structural constituent of ribosome | 37/849 | 2.43E-17 | 1.06E-14 |  |
| GO:0009055 | electron transfer activity | 24/849 | 2.73E-17 | 1.06E-14 |  |
| GO:0015078 | proton transmembrane transporter activity | 29/849 | 5.49E-16 | 1.42E-13 |  |
| GO:0050662 | coenzyme binding | 41/849 | 1.63E-13 | 1.81E-11 |  |
| GO:0031625 | ubiquitin protein ligase binding | 39/849 | 2.34E-11 | 2.02E-09 |  |
| GO:0003954 | NADH dehydrogenase activity | 12/849 | 1.20E-10 | 7.20E-09 |  |
| GO:0003779 | actin binding | 46/849 | 1.21E-10 | 7.20E-09 |  |
| GO:0051536 | iron-sulfur cluster binding | 17/849 | 1.59E-10 | 7.72E-09 |  |
| GO:0051540 | metal cluster binding RNA binding | 17/849 | 1.59E-10 | 7.72E-09 |  |
| GO:0008135 | translation factor activity | 16/849 | 2.02E-08 | 4.14E-07 |  |
| GO:0016829 | lyase activity | 22/849 | 9.87E-07 | 1.39E-05 |  |
| GO:0060590 | ATPase regulator activity | 9/849 | 1.05E-05 | 0.000118 |  |
| GO:0000287 | magnesium ion binding | 21/849 | 1.30E-05 | 0.000142 |  |
| GO:0004364 | glutathione transferase activity | 8/849 | 1.74E-05 | 0.000188 |  |
| GO:0048029 | monosaccharide binding | 12/849 | 7.34E-05 | 0.000687 |  |
| GO:0004298 | threonine-type endopeptidase activity | 6/849 | 8.95E-05 | 0.000809 |  |
| GO:0070003 | threonine-type peptidase activity | 6/849 | 8.95E-05 | 0.000809 |  |
| GO:0045182 | translation regulator activity | 8/849 | 0.001065 | 0.008271 |  |
| GO:0016289 | CoA hydrolase activity | 5/849 | 0.002049 | 0.014879 |  |
| GO:0016878 | acid-thiol ligase activity | 5/849 | 0.00246 | 0.017374 |  |
| GO:0019205 | nucleobase-containing compound kinase activity | 6/849 | 0.002652 | 0.018397 |  |
| GO:0005504 | fatty acid binding | 6/849 | 0.006276 | 0.040303 |  |
| GO:0016408 | C-acyltransferase activity | 4/849 | 0.006965 | 0.043643 |  |
| GO:0022829 | wide pore channel activity | 4/849 | 0.008265 | 0.049382 |  |
