# Supplementary material for: Ndufs1 Deficiency Aggravates the Mitochondrial Membrane Potential Dysfunction in Pressure Overload-Induced Myocardial Hypertrophy
Source: Oxid Med Cell Longev. 2021 Mar 3;2021:5545261. doi: 10.1155/2021/5545261 (PMC7952157; doi:10.1155/2021/5545261)
Supplement: Supplementary Materials — Additional file 1 Table S1: Gene Ontology enrichment terms for differentially expressed genes identified based on the scRNA-seq of pressure overload-induced myocardial hypertrophy. Additional file 2: Table S2: pathways correlated with pressure overload-induced myocardial hypertrophy based on time series-related GSEA. Additional file 3: Table S3: the oxidative phosphorylation score of single cardiomyocytes was calculated by GSVA. Additional file 4: Table S4: the cellular compartment-specific protein-protein interaction network of NDUFS1. Additional file 5: Table S5: biological process enrichment analyses of NDUFS1. [file 5545261.f1.zip › Table S2 GSEA results in the progression of myocardial hypertrophy based on time-serial analysis.doc]

**Table S2. GSEA results in the progression of myocardial hypertrophy based on time-serial analysis**

| **NAME** | **SIZE** | **ES** | **NES** | **NOM p-val** | | **FDR q-val** |
| --- | --- | --- | --- | --- | --- | --- |
| KEGG_CARDIAC_MUSCLE_CONTRACTION | 69 | 0.98 | 2.92 | 0.00 | 0.00 | |
| KEGG_HYPERTROPHIC_CARDIOMYOPATHY_HCM | 81 | 0.96 | 2.86 | 0.00 | 0.01 | |
| KEGG_CITRATE_CYCLE_TCA_CYCLE | 30 | 0.95 | 2.83 | 0.00 | 0.03 | |
| KEGG_OXIDATIVE_PHOSPHORYLATION | 110 | 0.97 | 2.80 | 0.00 | 0.04 | |
| KEGG_PARKINSONS_DISEASE | 103 | 0.97 | 2.77 | 0.00 | 0.04 | |
| KEGG_ALZHEIMERS_DISEASE | 147 | 0.96 | 2.71 | 0.00 | 0.09 | |
| KEGG_HUNTINGTONS_DISEASE | 158 | 0.96 | 2.71 | 0.00 | 0.07 | |
| KEGG_DILATED_CARDIOMYOPATHY | 87 | 0.96 | 2.68 | 0.00 | 0.08 | |
| KEGG_PYRUVATE_METABOLISM | 39 | 0.86 | 2.53 | 0.03 | 0.26 | |
| KEGG_PROTEASOME | 42 | 0.84 | 2.51 | 0.03 | 0.25 | |
| KEGG_PPAR_SIGNALING_PATHWAY | 68 | 0.84 | 2.44 | 0.02 | 0.31 | |
| KEGG_GLYOXYLATE_AND_DICARBOXYLATE_METABOLISM | 16 | 0.90 | 2.44 | 0.02 | 0.29 | |
| KEGG_PROPANOATE_METABOLISM | 31 | 0.82 | 2.36 | 0.04 | 0.34 | |
| KEGG_FATTY_ACID_METABOLISM | 39 | 0.78 | 2.32 | 0.05 | 0.36 | |
| KEGG_VALINE_LEUCINE_AND_ISOLEUCINE_DEGRADATION | 44 | 0.77 | 2.30 | 0.06 | 0.35 | |
| KEGG_LEUKOCYTE_TRANSENDOTHELIAL_MIGRATION | 111 | 0.79 | 2.28 | 0.03 | 0.36 | |
| KEGG_GLYCOLYSIS_GLUCONEOGENESIS | 56 | 0.79 | 2.27 | 0.05 | 0.34 | |
| KEGG_GLUTATHIONE_METABOLISM | 42 | 0.76 | 2.24 | 0.05 | 0.35 | |
| KEGG_TIGHT_JUNCTION | 125 | 0.79 | 2.24 | 0.02 | 0.33 | |
| KEGG_ARGININE_AND_PROLINE_METABOLISM | 50 | 0.72 | 2.21 | 0.05 | 0.35 | |
| KEGG_CALCIUM_SIGNALING_PATHWAY | 171 | 0.80 | 2.18 | 0.02 | 0.36 | |
| KEGG_PEROXISOME | 77 | 0.70 | 2.14 | 0.07 | 0.37 | |
| KEGG_PROXIMAL_TUBULE_BICARBONATE_RECLAMATION | 21 | 0.77 | 2.13 | 0.08 | 0.37 | |
| KEGG_AMINOACYL_TRNA_BIOSYNTHESIS | 41 | 0.68 | 2.02 | 0.10 | 0.45 | |
| KEGG_CYSTEINE_AND_METHIONINE_METABOLISM | 32 | 0.70 | 1.99 | 0.10 | 0.46 | |
| KEGG_BUTANOATE_METABOLISM | 33 | 0.69 | 1.96 | 0.10 | 0.47 | |
| KEGG_METABOLISM_OF_XENOBIOTICS_BY_CYTOCHROME_P450 | 39 | 0.64 | 1.93 | 0.11 | 0.48 | |
| KEGG_RIBOSOME | 57 | 0.64 | 1.92 | 0.10 | 0.48 | |
| KEGG_REGULATION_OF_ACTIN_CYTOSKELETON | 207 | 0.70 | 1.90 | 0.04 | 0.48 | |
| KEGG_BETA_ALANINE_METABOLISM | 22 | 0.64 | 1.88 | 0.12 | 0.49 | |
| KEGG_FOCAL_ADHESION | 192 | 0.70 | 1.87 | 0.05 | 0.48 | |
| KEGG_DRUG_METABOLISM_CYTOCHROME_P450 | 43 | 0.61 | 1.84 | 0.13 | 0.49 | |
| KEGG_PHENYLALANINE_METABOLISM | 18 | 0.63 | 1.80 | 0.11 | 0.51 | |
| KEGG_PROTEIN_EXPORT | 23 | 0.61 | 1.71 | 0.16 | 0.58 | |
| KEGG_ASTHMA | 22 | 0.56 | 1.63 | 0.15 | 0.63 | |
| KEGG_ANTIGEN_PROCESSING_AND_PRESENTATION | 52 | 0.53 | 1.62 | 0.18 | 0.62 | |
| KEGG_VIRAL_MYOCARDITIS | 54 | 0.53 | 1.57 | 0.20 | 0.66 | |
| KEGG_ARRHYTHMOGENIC_RIGHT_VENTRICULAR_CARDIOMYOPATHY_ARVC | 74 | 0.53 | 1.56 | 0.20 | 0.65 | |
| KEGG_FRUCTOSE_AND_MANNOSE_METABOLISM | 33 | 0.54 | 1.56 | 0.21 | 0.64 | |
| KEGG_BIOSYNTHESIS_OF_UNSATURATED_FATTY_ACIDS | 21 | 0.55 | 1.55 | 0.18 | 0.63 | |
| KEGG_PATHOGENIC_ESCHERICHIA_COLI_INFECTION | 52 | 0.50 | 1.50 | 0.21 | 0.66 | |
| KEGG_LYSINE_DEGRADATION | 41 | 0.49 | 1.49 | 0.22 | 0.66 | |
| KEGG_TRYPTOPHAN_METABOLISM | 39 | 0.51 | 1.44 | 0.24 | 0.69 | |
| KEGG_AUTOIMMUNE_THYROID_DISEASE | 35 | 0.47 | 1.38 | 0.25 | 0.73 | |
| KEGG_VIBRIO_CHOLERAE_INFECTION | 52 | 0.46 | 1.38 | 0.25 | 0.72 | |
| KEGG_TYROSINE_METABOLISM | 38 | 0.46 | 1.37 | 0.25 | 0.71 | |
| KEGG_GLYCEROLIPID_METABOLISM | 48 | 0.46 | 1.36 | 0.25 | 0.70 | |
| KEGG_PRION_DISEASES | 31 | 0.47 | 1.36 | 0.24 | 0.69 | |
| KEGG_PENTOSE_PHOSPHATE_PATHWAY | 25 | 0.48 | 1.34 | 0.25 | 0.69 | |
| KEGG_ALLOGRAFT_REJECTION | 24 | 0.47 | 1.34 | 0.25 | 0.68 | |
| KEGG_MATURITY_ONSET_DIABETES_OF_THE_YOUNG | 24 | 0.46 | 1.33 | 0.25 | 0.67 | |
| KEGG_RNA_POLYMERASE | 25 | 0.47 | 1.33 | 0.25 | 0.66 | |
| KEGG_RNA_DEGRADATION | 55 | 0.44 | 1.28 | 0.29 | 0.70 | |
| KEGG_OLFACTORY_TRANSDUCTION | 278 | 0.49 | 1.27 | 0.26 | 0.69 | |
| KEGG_REGULATION_OF_AUTOPHAGY | 30 | 0.44 | 1.27 | 0.30 | 0.68 | |
| KEGG_ALANINE_ASPARTATE_AND_GLUTAMATE_METABOLISM | 30 | 0.40 | 1.18 | 0.30 | 0.75 | |
| KEGG_STARCH_AND_SUCROSE_METABOLISM | 34 | 0.40 | 1.14 | 0.33 | 0.77 | |
| KEGG_RIBOFLAVIN_METABOLISM | 15 | 0.42 | 1.14 | 0.30 | 0.77 | |
| KEGG_PORPHYRIN_AND_CHLOROPHYLL_METABOLISM | 28 | 0.37 | 1.12 | 0.30 | 0.77 | |
| KEGG_GALACTOSE_METABOLISM | 26 | 0.39 | 1.12 | 0.30 | 0.75 | |
| KEGG_SNARE_INTERACTIONS_IN_VESICULAR_TRANSPORT | 34 | 0.37 | 1.11 | 0.33 | 0.75 | |
| KEGG_EPITHELIAL_CELL_SIGNALING_IN_HELICOBACTER_PYLORI_INFECTION | 65 | 0.37 | 1.09 | 0.38 | 0.76 | |
| KEGG_PYRIMIDINE_METABOLISM | 91 | 0.36 | 1.07 | 0.39 | 0.77 | |
| KEGG_GRAFT_VERSUS_HOST_DISEASE | 22 | 0.37 | 1.03 | 0.36 | 0.79 | |
| KEGG_TYPE_I_DIABETES_MELLITUS | 29 | 0.35 | 1.00 | 0.37 | 0.81 | |
| KEGG_TASTE_TRANSDUCTION | 42 | 0.35 | 1.00 | 0.39 | 0.80 | |
| KEGG_COMPLEMENT_AND_COAGULATION_CASCADES | 64 | 0.34 | 0.99 | 0.42 | 0.80 | |
| KEGG_SYSTEMIC_LUPUS_ERYTHEMATOSUS | 78 | 0.34 | 0.98 | 0.43 | 0.80 | |
| KEGG_PENTOSE_AND_GLUCURONATE_INTERCONVERSIONS | 15 | 0.36 | 0.96 | 0.39 | 0.80 | |
| KEGG_PURINE_METABOLISM | 151 | 0.34 | 0.96 | 0.48 | 0.79 | |
| KEGG_NEUROACTIVE_LIGAND_RECEPTOR_INTERACTION | 258 | 0.37 | 0.96 | 0.48 | 0.78 | |
| KEGG_ARACHIDONIC_ACID_METABOLISM | 51 | 0.33 | 0.94 | 0.43 | 0.79 | |
| KEGG_OOCYTE_MEIOSIS | 105 | 0.33 | 0.94 | 0.45 | 0.78 | |
| KEGG_RETINOL_METABOLISM | 40 | 0.32 | 0.92 | 0.43 | 0.79 | |
| KEGG_STEROID_HORMONE_BIOSYNTHESIS | 34 | 0.31 | 0.92 | 0.40 | 0.78 | |
| KEGG_ADHERENS_JUNCTION | 72 | 0.30 | 0.90 | 0.44 | 0.79 | |
| KEGG_HEMATOPOIETIC_CELL_LINEAGE | 77 | 0.31 | 0.90 | 0.47 | 0.78 | |
| KEGG_RENAL_CELL_CARCINOMA | 68 | 0.31 | 0.89 | 0.46 | 0.78 | |
| KEGG_LYSOSOME | 116 | 0.30 | 0.89 | 0.48 | 0.77 | |
| KEGG_NITROGEN_METABOLISM | 22 | 0.31 | 0.86 | 0.45 | 0.80 | |
| KEGG_AMINO_SUGAR_AND_NUCLEOTIDE_SUGAR_METABOLISM | 44 | 0.29 | 0.85 | 0.46 | 0.79 | |
| KEGG_CELL_ADHESION_MOLECULES_CAMS | 112 | 0.28 | 0.85 | 0.50 | 0.79 | |
| KEGG_PRIMARY_IMMUNODEFICIENCY | 33 | 0.29 | 0.83 | 0.50 | 0.79 | |
| KEGG_INTESTINAL_IMMUNE_NETWORK_FOR_IGA_PRODUCTION | 39 | 0.29 | 0.81 | 0.51 | 0.80 | |
| KEGG_INOSITOL_PHOSPHATE_METABOLISM | 54 | 0.27 | 0.81 | 0.49 | 0.80 | |
| KEGG_GAP_JUNCTION | 83 | 0.27 | 0.80 | 0.54 | 0.80 | |
| KEGG_INSULIN_SIGNALING_PATHWAY | 132 | 0.29 | 0.78 | 0.61 | 0.82 | |
| KEGG_LINOLEIC_ACID_METABOLISM | 24 | 0.28 | 0.77 | 0.49 | 0.81 | |
| KEGG_CYTOKINE_CYTOKINE_RECEPTOR_INTERACTION | 234 | 0.29 | 0.76 | 0.65 | 0.82 | |
| KEGG_AMYOTROPHIC_LATERAL_SCLEROSIS_ALS | 51 | 0.25 | 0.75 | 0.54 | 0.82 | |
| KEGG_UBIQUITIN_MEDIATED_PROTEOLYSIS | 124 | 0.26 | 0.74 | 0.60 | 0.81 | |
| KEGG_ALPHA_LINOLENIC_ACID_METABOLISM | 18 | 0.26 | 0.73 | 0.57 | 0.82 | |
| KEGG_P53_SIGNALING_PATHWAY | 62 | 0.23 | 0.69 | 0.59 | 0.86 | |
| KEGG_GLYCOSAMINOGLYCAN_BIOSYNTHESIS_CHONDROITIN_SULFATE | 22 | 0.24 | 0.68 | 0.58 | 0.86 | |
| KEGG_MTOR_SIGNALING_PATHWAY | 51 | 0.23 | 0.68 | 0.60 | 0.85 | |
| KEGG_LONG_TERM_POTENTIATION | 65 | 0.23 | 0.67 | 0.60 | 0.85 | |
| KEGG_BASAL_CELL_CARCINOMA | 55 | 0.22 | 0.67 | 0.60 | 0.84 | |
| KEGG_GLYCINE_SERINE_AND_THREONINE_METABOLISM | 31 | 0.22 | 0.66 | 0.62 | 0.85 | |
| KEGG_RENIN_ANGIOTENSIN_SYSTEM | 17 | 0.23 | 0.64 | 0.65 | 0.85 | |
| KEGG_CELL_CYCLE | 118 | 0.22 | 0.63 | 0.66 | 0.86 | |
| KEGG_TERPENOID_BACKBONE_BIOSYNTHESIS | 15 | 0.23 | 0.61 | 0.69 | 0.87 | |
| KEGG_ADIPOCYTOKINE_SIGNALING_PATHWAY | 67 | 0.20 | 0.59 | 0.68 | 0.89 | |
| KEGG_ECM_RECEPTOR_INTERACTION | 82 | 0.19 | 0.57 | 0.71 | 0.90 | |
| KEGG_FC_GAMMA_R_MEDIATED_PHAGOCYTOSIS | 92 | 0.20 | 0.56 | 0.73 | 0.91 | |
| KEGG_ALDOSTERONE_REGULATED_SODIUM_REABSORPTION | 41 | 0.18 | 0.56 | 0.69 | 0.91 | |
| KEGG_NOD_LIKE_RECEPTOR_SIGNALING_PATHWAY | 52 | 0.19 | 0.56 | 0.71 | 0.90 | |
| KEGG_VASCULAR_SMOOTH_MUSCLE_CONTRACTION | 109 | 0.18 | 0.51 | 0.76 | 0.94 | |
| KEGG_PROGESTERONE_MEDIATED_OOCYTE_MATURATION | 80 | 0.17 | 0.50 | 0.76 | 0.94 | |
| KEGG_CYTOSOLIC_DNA_SENSING_PATHWAY | 48 | 0.17 | 0.49 | 0.78 | 0.95 | |
| KEGG_O_GLYCAN_BIOSYNTHESIS | 29 | 0.17 | 0.49 | 0.78 | 0.94 | |
| KEGG_GLYCOSPHINGOLIPID_BIOSYNTHESIS_LACTO_AND_NEOLACTO_SERIES | 22 | 0.17 | 0.49 | 0.84 | 0.94 | |
| KEGG_NICOTINATE_AND_NICOTINAMIDE_METABOLISM | 23 | 0.17 | 0.48 | 0.80 | 0.93 | |
| KEGG_VASOPRESSIN_REGULATED_WATER_REABSORPTION | 43 | 0.16 | 0.46 | 0.80 | 0.95 | |
| KEGG_TGF_BETA_SIGNALING_PATHWAY | 80 | 0.15 | 0.45 | 0.81 | 0.96 | |
| KEGG_VEGF_SIGNALING_PATHWAY | 72 | 0.15 | 0.44 | 0.82 | 0.95 | |
| KEGG_GLIOMA | 59 | 0.14 | 0.43 | 0.83 | 0.96 | |
| KEGG_ETHER_LIPID_METABOLISM | 31 | 0.15 | 0.42 | 0.84 | 0.96 | |
| KEGG_JAK_STAT_SIGNALING_PATHWAY | 141 | 0.15 | 0.42 | 0.88 | 0.95 | |
| KEGG_NATURAL_KILLER_CELL_MEDIATED_CYTOTOXICITY | 104 | 0.14 | 0.41 | 0.85 | 0.96 | |
| KEGG_DRUG_METABOLISM_OTHER_ENZYMES | 35 | 0.13 | 0.38 | 0.89 | 0.98 | |
| KEGG_B_CELL_RECEPTOR_SIGNALING_PATHWAY | 73 | 0.13 | 0.38 | 0.85 | 0.97 | |
| KEGG_PROSTATE_CANCER | 86 | 0.13 | 0.37 | 0.88 | 0.97 | |
| KEGG_ENDOCYTOSIS | 168 | 0.13 | 0.37 | 0.90 | 0.96 | |
| KEGG_GNRH_SIGNALING_PATHWAY | 93 | 0.11 | 0.32 | 0.93 | 1.00 | |
| KEGG_LEISHMANIA_INFECTION | 63 | 0.11 | 0.32 | 0.92 | 0.99 | |
| KEGG_HISTIDINE_METABOLISM | 28 | 0.11 | 0.31 | 0.96 | 0.99 | |
| KEGG_PATHWAYS_IN_CANCER | 314 | 0.11 | 0.29 | 0.98 | 1.00 | |
| KEGG_AXON_GUIDANCE | 129 | 0.09 | 0.26 | 0.97 | 1.00 | |
| KEGG_MELANOGENESIS | 96 | 0.09 | 0.26 | 0.97 | 1.00 | |
| KEGG_WNT_SIGNALING_PATHWAY | 145 | 0.08 | 0.23 | 0.98 | 1.00 | |
| KEGG_LONG_TERM_DEPRESSION | 67 | 0.06 | 0.19 | 0.99 | 1.00 | |
| KEGG_CHEMOKINE_SIGNALING_PATHWAY | 165 | 0.04 | 0.10 | 1.00 | 1.00 | |
