# Supplementary material for: Ndufs1 Deficiency Aggravates the Mitochondrial Membrane Potential Dysfunction in Pressure Overload-Induced Myocardial Hypertrophy
Source: Oxid Med Cell Longev. 2021 Mar 3;2021:5545261. doi: 10.1155/2021/5545261 (PMC7952157; doi:10.1155/2021/5545261)
Supplement: Supplementary Materials — Additional file 1 Table S1: Gene Ontology enrichment terms for differentially expressed genes identified based on the scRNA-seq of pressure overload-induced myocardial hypertrophy. Additional file 2: Table S2: pathways correlated with pressure overload-induced myocardial hypertrophy based on time series-related GSEA. Additional file 3: Table S3: the oxidative phosphorylation score of single cardiomyocytes was calculated by GSVA. Additional file 4: Table S4: the cellular compartment-specific protein-protein interaction network of NDUFS1. Additional file 5: Table S5: biological process enrichment analyses of NDUFS1. [file 5545261.f1.zip › Table S3 GSVA oxidative phosphorylation score.doc]

**Table S3 GSVA oxidative phosphorylation score**

| **Term** | **Group** | **Score** |
| --- | --- | --- |
| Oxidative phosphorylation score | Sham | -0.11612 |
| Oxidative phosphorylation score | Sham | 0.542655 |
| Oxidative phosphorylation score | Sham | 0.251567 |
| Oxidative phosphorylation score | Sham | 0.293352 |
| Oxidative phosphorylation score | Sham | 0.395047 |
| Oxidative phosphorylation score | Sham | 0.3236 |
| Oxidative phosphorylation score | Sham | 0.338889 |
| Oxidative phosphorylation score | Sham | 0.480159 |
| Oxidative phosphorylation score | Sham | 0.417334 |
| Oxidative phosphorylation score | Sham | -0.31498 |
| Oxidative phosphorylation score | Sham | -0.57301 |
| Oxidative phosphorylation score | Sham | 0.518367 |
| Oxidative phosphorylation score | Sham | 0.428622 |
| Oxidative phosphorylation score | Sham | -0.22345 |
| Oxidative phosphorylation score | Sham | 0.517833 |
| Oxidative phosphorylation score | Sham | 0.403587 |
| Oxidative phosphorylation score | Sham | 0.338009 |
| Oxidative phosphorylation score | Sham | 0.53171 |
| Oxidative phosphorylation score | Sham | 0.092008 |
| Oxidative phosphorylation score | Sham | 0.506123 |
| Oxidative phosphorylation score | Sham | 0.452632 |
| Oxidative phosphorylation score | Sham | 0.261852 |
| Oxidative phosphorylation score | Sham | -0.66048 |
| Oxidative phosphorylation score | Sham | 0.523749 |
| Oxidative phosphorylation score | Sham | -0.70334 |
| Oxidative phosphorylation score | Sham | -0.68742 |
| Oxidative phosphorylation score | Sham | -0.07643 |
| Oxidative phosphorylation score | Sham | 0.083358 |
| Oxidative phosphorylation score | Sham | 0.191777 |
| Oxidative phosphorylation score | Sham | -0.66712 |
| Oxidative phosphorylation score | Sham | -0.16173 |
| Oxidative phosphorylation score | Sham | -0.46551 |
| Oxidative phosphorylation score | Sham | 0.065914 |
| Oxidative phosphorylation score | Sham | 0.024245 |
| Oxidative phosphorylation score | Sham | -0.58513 |
| Oxidative phosphorylation score | Sham | -0.58125 |
| Oxidative phosphorylation score | Sham | -0.27362 |
| Oxidative phosphorylation score | Sham | 0.023827 |
| Oxidative phosphorylation score | Sham | -0.511 |
| Oxidative phosphorylation score | Sham | 0.60343 |
| Oxidative phosphorylation score | Sham | 0.372714 |
| Oxidative phosphorylation score | Sham | 0.493315 |
| Oxidative phosphorylation score | Sham | 0.31091 |
| Oxidative phosphorylation score | Sham | 0.281996 |
| Oxidative phosphorylation score | Sham | 0.260901 |
| Oxidative phosphorylation score | Sham | 0.521673 |
| Oxidative phosphorylation score | Sham | -0.55941 |
| Oxidative phosphorylation score | Sham | 0.074762 |
| Oxidative phosphorylation score | Sham | 0.40238 |
| Oxidative phosphorylation score | Sham | 0.512973 |
| Oxidative phosphorylation score | Sham | 0.591562 |
| Oxidative phosphorylation score | Sham | -0.71235 |
| Oxidative phosphorylation score | Sham | -0.14285 |
| Oxidative phosphorylation score | Sham | 0.491451 |
| Oxidative phosphorylation score | Sham | 0.487498 |
| Oxidative phosphorylation score | Sham | -0.70316 |
| Oxidative phosphorylation score | Sham | 0.494659 |
| Oxidative phosphorylation score | Sham | -0.53404 |
| Oxidative phosphorylation score | Sham | 0.305244 |
| Oxidative phosphorylation score | Sham | 0.535307 |
| Oxidative phosphorylation score | Sham | -0.41539 |
| Oxidative phosphorylation score | Sham | 0.564415 |
| Oxidative phosphorylation score | Sham | 0.435184 |
| Oxidative phosphorylation score | Sham | 0.510169 |
| Oxidative phosphorylation score | Sham | 0.459086 |
| Oxidative phosphorylation score | Sham | 0.488413 |
| Oxidative phosphorylation score | Sham | -0.67023 |
| Oxidative phosphorylation score | Sham | -0.00792 |
| Oxidative phosphorylation score | Sham | -0.22727 |
| Oxidative phosphorylation score | Sham | -0.32967 |
| Oxidative phosphorylation score | Sham | 0.009605 |
| Oxidative phosphorylation score | Sham | -0.75736 |
| Oxidative phosphorylation score | Sham | 0.315692 |
| Oxidative phosphorylation score | Sham | 0.552963 |
| Oxidative phosphorylation score | Sham | 0.252655 |
| Oxidative phosphorylation score | Sham | -0.14624 |
| Oxidative phosphorylation score | Sham | 0.052966 |
| Oxidative phosphorylation score | Sham | -0.10182 |
| Oxidative phosphorylation score | Sham | -0.02145 |
| Oxidative phosphorylation score | Sham | -0.65024 |
| Oxidative phosphorylation score | Sham | -0.00791 |
| Oxidative phosphorylation score | Sham | 0.212332 |
| Oxidative phosphorylation score | Sham | -0.47808 |
| Oxidative phosphorylation score | Sham | 0.147545 |
| Oxidative phosphorylation score | Sham | -0.66231 |
| Oxidative phosphorylation score | Sham | -0.58248 |
| Oxidative phosphorylation score | Sham | -0.43257 |
| Oxidative phosphorylation score | Sham | -0.36792 |
| Oxidative phosphorylation score | Sham | -0.11146 |
| Oxidative phosphorylation score | Sham | -0.20465 |
| Oxidative phosphorylation score | TAC D3 | 0.498736 |
| Oxidative phosphorylation score | TAC D3 | 0.514779 |
| Oxidative phosphorylation score | TAC D3 | 0.442473 |
| Oxidative phosphorylation score | TAC D3 | 0.22569 |
| Oxidative phosphorylation score | TAC D3 | -0.41735 |
| Oxidative phosphorylation score | TAC D3 | 0.285925 |
| Oxidative phosphorylation score | TAC D3 | 0.527476 |
| Oxidative phosphorylation score | TAC D3 | -0.21343 |
| Oxidative phosphorylation score | TAC D3 | 0.032604 |
| Oxidative phosphorylation score | TAC D3 | 0.367766 |
| Oxidative phosphorylation score | TAC D3 | 0.419001 |
| Oxidative phosphorylation score | TAC D3 | -0.58028 |
| Oxidative phosphorylation score | TAC D3 | 0.579244 |
| Oxidative phosphorylation score | TAC D3 | -0.25588 |
| Oxidative phosphorylation score | TAC D3 | -0.60723 |
| Oxidative phosphorylation score | TAC D3 | 0.197811 |
| Oxidative phosphorylation score | TAC D3 | 0.440603 |
| Oxidative phosphorylation score | TAC D3 | 0.218295 |
| Oxidative phosphorylation score | TAC D3 | 0.28368 |
| Oxidative phosphorylation score | TAC D3 | 0.542636 |
| Oxidative phosphorylation score | TAC D3 | 0.496291 |
| Oxidative phosphorylation score | TAC D3 | 0.444263 |
| Oxidative phosphorylation score | TAC D3 | -0.3274 |
| Oxidative phosphorylation score | TAC D3 | 0.605187 |
| Oxidative phosphorylation score | TAC D3 | 0.299989 |
| Oxidative phosphorylation score | TAC D3 | 0.204257 |
| Oxidative phosphorylation score | TAC D3 | 0.27526 |
| Oxidative phosphorylation score | TAC D3 | 0.427114 |
| Oxidative phosphorylation score | TAC D3 | 0.390135 |
| Oxidative phosphorylation score | TAC D3 | 0.012671 |
| Oxidative phosphorylation score | TAC D3 | 0.549115 |
| Oxidative phosphorylation score | TAC D3 | 0.221732 |
| Oxidative phosphorylation score | TAC D3 | 0.541128 |
| Oxidative phosphorylation score | TAC D3 | 0.641396 |
| Oxidative phosphorylation score | TAC D3 | 0.317755 |
| Oxidative phosphorylation score | TAC D3 | 0.57709 |
| Oxidative phosphorylation score | TAC D3 | 0.524287 |
| Oxidative phosphorylation score | TAC D3 | 0.483776 |
| Oxidative phosphorylation score | TAC D3 | 0.487749 |
| Oxidative phosphorylation score | TAC D3 | 0.620099 |
| Oxidative phosphorylation score | TAC D3 | 0.151723 |
| Oxidative phosphorylation score | TAC D3 | -0.05123 |
| Oxidative phosphorylation score | TAC D3 | 0.498076 |
| Oxidative phosphorylation score | TAC D3 | 0.086337 |
| Oxidative phosphorylation score | TAC D3 | 0.623021 |
| Oxidative phosphorylation score | TAC D3 | 0.372241 |
| Oxidative phosphorylation score | TAC D3 | 0.340495 |
| Oxidative phosphorylation score | TAC D3 | 0.596975 |
| Oxidative phosphorylation score | TAC D3 | -0.43091 |
| Oxidative phosphorylation score | TAC D3 | 0.578467 |
| Oxidative phosphorylation score | TAC D3 | 0.497047 |
| Oxidative phosphorylation score | TAC D3 | 0.483456 |
| Oxidative phosphorylation score | TAC D3 | 0.571695 |
| Oxidative phosphorylation score | TAC D3 | 0.455523 |
| Oxidative phosphorylation score | TAC D3 | 0.285178 |
| Oxidative phosphorylation score | TAC D3 | 0.5891 |
| Oxidative phosphorylation score | TAC D3 | 0.414894 |
| Oxidative phosphorylation score | TAC D3 | 0.040775 |
| Oxidative phosphorylation score | TAC D3 | 0.54844 |
| Oxidative phosphorylation score | TAC D3 | 0.429719 |
| Oxidative phosphorylation score | TAC D3 | 0.005265 |
| Oxidative phosphorylation score | TAC D3 | 0.555014 |
| Oxidative phosphorylation score | TAC D3 | 0.438376 |
| Oxidative phosphorylation score | TAC D3 | 0.518555 |
| Oxidative phosphorylation score | TAC D3 | 0.356271 |
| Oxidative phosphorylation score | TAC D3 | 0.410498 |
| Oxidative phosphorylation score | TAC D3 | -0.02136 |
| Oxidative phosphorylation score | TAC D3 | -0.23523 |
| Oxidative phosphorylation score | TAC D3 | -0.25384 |
| Oxidative phosphorylation score | TAC D3 | -0.60047 |
| Oxidative phosphorylation score | TAC D3 | -0.52921 |
| Oxidative phosphorylation score | TAC W1 | -0.54568 |
| Oxidative phosphorylation score | TAC W1 | -0.57212 |
| Oxidative phosphorylation score | TAC W1 | -0.5999 |
| Oxidative phosphorylation score | TAC W1 | -0.12875 |
| Oxidative phosphorylation score | TAC W1 | -0.54882 |
| Oxidative phosphorylation score | TAC W1 | 0.28451 |
| Oxidative phosphorylation score | TAC W1 | -0.5509 |
| Oxidative phosphorylation score | TAC W1 | -0.46376 |
| Oxidative phosphorylation score | TAC W1 | -0.39485 |
| Oxidative phosphorylation score | TAC W1 | -0.22089 |
| Oxidative phosphorylation score | TAC W1 | -0.4993 |
| Oxidative phosphorylation score | TAC W1 | -0.0622 |
| Oxidative phosphorylation score | TAC W1 | -0.39266 |
| Oxidative phosphorylation score | TAC W1 | -0.58398 |
| Oxidative phosphorylation score | TAC W1 | -0.56525 |
| Oxidative phosphorylation score | TAC W1 | -0.66174 |
| Oxidative phosphorylation score | TAC W1 | 0.222694 |
| Oxidative phosphorylation score | TAC W1 | -0.59645 |
| Oxidative phosphorylation score | TAC W1 | -0.45456 |
| Oxidative phosphorylation score | TAC W1 | -0.27913 |
| Oxidative phosphorylation score | TAC W1 | -0.62918 |
| Oxidative phosphorylation score | TAC W1 | -0.17785 |
| Oxidative phosphorylation score | TAC W1 | -0.60482 |
| Oxidative phosphorylation score | TAC W1 | -0.63411 |
| Oxidative phosphorylation score | TAC W1 | 0.068336 |
| Oxidative phosphorylation score | TAC W1 | -0.6274 |
| Oxidative phosphorylation score | TAC W1 | -0.11878 |
| Oxidative phosphorylation score | TAC W1 | -0.56463 |
| Oxidative phosphorylation score | TAC W1 | -0.58969 |
| Oxidative phosphorylation score | TAC W1 | -0.64412 |
| Oxidative phosphorylation score | TAC W1 | -0.66867 |
| Oxidative phosphorylation score | TAC W1 | -0.66618 |
| Oxidative phosphorylation score | TAC W1 | -0.13392 |
| Oxidative phosphorylation score | TAC W1 | -0.32194 |
| Oxidative phosphorylation score | TAC W1 | 0.133279 |
| Oxidative phosphorylation score | TAC W1 | -0.39767 |
| Oxidative phosphorylation score | TAC W1 | -0.29749 |
| Oxidative phosphorylation score | TAC W1 | -0.20026 |
| Oxidative phosphorylation score | TAC W1 | 0.133741 |
| Oxidative phosphorylation score | TAC W1 | -0.53178 |
| Oxidative phosphorylation score | TAC W1 | -0.16255 |
| Oxidative phosphorylation score | TAC W1 | -0.56847 |
| Oxidative phosphorylation score | TAC W1 | -0.41073 |
| Oxidative phosphorylation score | TAC W1 | -0.62308 |
| Oxidative phosphorylation score | TAC W1 | -0.56284 |
| Oxidative phosphorylation score | TAC W1 | 0.030557 |
| Oxidative phosphorylation score | TAC W1 | -0.50333 |
| Oxidative phosphorylation score | TAC W1 | -0.37876 |
| Oxidative phosphorylation score | TAC W1 | -0.06988 |
| Oxidative phosphorylation score | TAC W1 | -0.04487 |
| Oxidative phosphorylation score | TAC W1 | -0.31621 |
| Oxidative phosphorylation score | TAC W1 | 0.430906 |
| Oxidative phosphorylation score | TAC W1 | -0.23778 |
| Oxidative phosphorylation score | TAC W1 | 0.080928 |
| Oxidative phosphorylation score | TAC W1 | 0.1855 |
| Oxidative phosphorylation score | TAC W1 | -0.36376 |
| Oxidative phosphorylation score | TAC W1 | 0.059758 |
| Oxidative phosphorylation score | TAC W1 | -0.39153 |
| Oxidative phosphorylation score | TAC W1 | -0.14309 |
| Oxidative phosphorylation score | TAC W1 | 0.67835 |
| Oxidative phosphorylation score | TAC W1 | -0.13719 |
| Oxidative phosphorylation score | TAC W1 | -0.43057 |
| Oxidative phosphorylation score | TAC W1 | -0.19437 |
| Oxidative phosphorylation score | TAC W1 | -0.32546 |
| Oxidative phosphorylation score | TAC W1 | -0.12589 |
| Oxidative phosphorylation score | TAC W1 | 0.308525 |
| Oxidative phosphorylation score | TAC W1 | -0.10689 |
| Oxidative phosphorylation score | TAC W1 | -0.39824 |
| Oxidative phosphorylation score | TAC W1 | -0.51706 |
| Oxidative phosphorylation score | TAC W1 | 0.36931 |
| Oxidative phosphorylation score | TAC W1 | 0.191363 |
| Oxidative phosphorylation score | TAC W1 | -0.27286 |
| Oxidative phosphorylation score | TAC W1 | -0.39128 |
| Oxidative phosphorylation score | TAC W1 | -0.43349 |
| Oxidative phosphorylation score | TAC W1 | -0.02868 |
| Oxidative phosphorylation score | TAC W1 | -0.30999 |
| Oxidative phosphorylation score | TAC W1 | 0.49913 |
| Oxidative phosphorylation score | TAC W1 | -0.3112 |
| Oxidative phosphorylation score | TAC W1 | 0.387802 |
| Oxidative phosphorylation score | TAC W1 | -0.52543 |
| Oxidative phosphorylation score | TAC W1 | 0.25296 |
| Oxidative phosphorylation score | TAC W1 | -0.28979 |
| Oxidative phosphorylation score | TAC W1 | -0.54663 |
| Oxidative phosphorylation score | TAC W1 | 0.019892 |
| Oxidative phosphorylation score | TAC W1 | 0.204859 |
| Oxidative phosphorylation score | TAC W2 | 0.328271 |
| Oxidative phosphorylation score | TAC W2 | 0.50476 |
| Oxidative phosphorylation score | TAC W2 | 0.368113 |
| Oxidative phosphorylation score | TAC W2 | 0.592895 |
| Oxidative phosphorylation score | TAC W2 | 0.574764 |
| Oxidative phosphorylation score | TAC W2 | 0.435435 |
| Oxidative phosphorylation score | TAC W2 | 0.565111 |
| Oxidative phosphorylation score | TAC W2 | 0.333879 |
| Oxidative phosphorylation score | TAC W2 | 0.528181 |
| Oxidative phosphorylation score | TAC W2 | 0.406056 |
| Oxidative phosphorylation score | TAC W2 | 0.604624 |
| Oxidative phosphorylation score | TAC W2 | 0.480714 |
| Oxidative phosphorylation score | TAC W2 | 0.404752 |
| Oxidative phosphorylation score | TAC W2 | 0.450229 |
| Oxidative phosphorylation score | TAC W2 | 0.265894 |
| Oxidative phosphorylation score | TAC W2 | 0.458379 |
| Oxidative phosphorylation score | TAC W2 | 0.603325 |
| Oxidative phosphorylation score | TAC W2 | -0.23169 |
| Oxidative phosphorylation score | TAC W2 | 0.413647 |
| Oxidative phosphorylation score | TAC W2 | 0.543843 |
| Oxidative phosphorylation score | TAC W2 | 0.194273 |
| Oxidative phosphorylation score | TAC W2 | 0.42268 |
| Oxidative phosphorylation score | TAC W2 | 0.617851 |
| Oxidative phosphorylation score | TAC W2 | -0.65535 |
| Oxidative phosphorylation score | TAC W2 | 0.455202 |
| Oxidative phosphorylation score | TAC W2 | -0.48561 |
| Oxidative phosphorylation score | TAC W2 | 0.126999 |
| Oxidative phosphorylation score | TAC W2 | 0.485456 |
| Oxidative phosphorylation score | TAC W2 | 0.223148 |
| Oxidative phosphorylation score | TAC W2 | 0.383617 |
| Oxidative phosphorylation score | TAC W2 | -0.22735 |
| Oxidative phosphorylation score | TAC W2 | 0.595528 |
| Oxidative phosphorylation score | TAC W2 | -0.0919 |
| Oxidative phosphorylation score | TAC W2 | 0.583389 |
| Oxidative phosphorylation score | TAC W2 | 0.04824 |
| Oxidative phosphorylation score | TAC W2 | 0.248187 |
| Oxidative phosphorylation score | TAC W2 | 0.31958 |
| Oxidative phosphorylation score | TAC W2 | 0.336663 |
| Oxidative phosphorylation score | TAC W2 | 0.237049 |
| Oxidative phosphorylation score | TAC W2 | 0.554153 |
| Oxidative phosphorylation score | TAC W2 | -0.71781 |
| Oxidative phosphorylation score | TAC W2 | -0.25232 |
| Oxidative phosphorylation score | TAC W2 | -0.72499 |
| Oxidative phosphorylation score | TAC W2 | 0.345808 |
| Oxidative phosphorylation score | TAC W2 | -0.22937 |
| Oxidative phosphorylation score | TAC W2 | 0.189372 |
| Oxidative phosphorylation score | TAC W2 | 0.574787 |
| Oxidative phosphorylation score | TAC W2 | 0.261406 |
| Oxidative phosphorylation score | TAC W2 | 0.21568 |
| Oxidative phosphorylation score | TAC W2 | 0.650037 |
| Oxidative phosphorylation score | TAC W2 | 0.340736 |
| Oxidative phosphorylation score | TAC W2 | -0.76006 |
| Oxidative phosphorylation score | TAC W2 | 0.16267 |
| Oxidative phosphorylation score | TAC W2 | -0.41002 |
| Oxidative phosphorylation score | TAC W2 | 0.620043 |
| Oxidative phosphorylation score | TAC W2 | -0.44108 |
| Oxidative phosphorylation score | TAC W2 | 0.529587 |
| Oxidative phosphorylation score | TAC W2 | -0.22849 |
| Oxidative phosphorylation score | TAC W2 | 0.231071 |
| Oxidative phosphorylation score | TAC W2 | -0.74663 |
| Oxidative phosphorylation score | TAC W2 | -0.66055 |
| Oxidative phosphorylation score | TAC W2 | 0.462015 |
| Oxidative phosphorylation score | TAC W2 | -0.75381 |
| Oxidative phosphorylation score | TAC W2 | -0.7333 |
| Oxidative phosphorylation score | TAC W2 | -0.38986 |
| Oxidative phosphorylation score | TAC W2 | -0.62419 |
| Oxidative phosphorylation score | TAC W2 | 0.639407 |
| Oxidative phosphorylation score | TAC W2 | -0.74966 |
| Oxidative phosphorylation score | TAC W2 | -0.64628 |
| Oxidative phosphorylation score | TAC W2 | -0.57013 |
| Oxidative phosphorylation score | TAC W2 | 0.074647 |
| Oxidative phosphorylation score | TAC W2 | 0.493435 |
| Oxidative phosphorylation score | TAC W2 | -0.60417 |
| Oxidative phosphorylation score | TAC W2 | -0.71803 |
| Oxidative phosphorylation score | TAC W2 | -0.20998 |
| Oxidative phosphorylation score | TAC W2 | -0.53016 |
| Oxidative phosphorylation score | TAC W2 | 0.578015 |
| Oxidative phosphorylation score | TAC W2 | -0.65888 |
| Oxidative phosphorylation score | TAC W2 | -0.75446 |
| Oxidative phosphorylation score | TAC W2 | -0.57979 |
| Oxidative phosphorylation score | TAC W2 | -0.46093 |
| Oxidative phosphorylation score | TAC W2 | -0.65274 |
| Oxidative phosphorylation score | TAC W2 | -0.67352 |
| Oxidative phosphorylation score | TAC W2 | 0.273882 |
| Oxidative phosphorylation score | TAC W4 | 0.592251 |
| Oxidative phosphorylation score | TAC W4 | 0.015849 |
| Oxidative phosphorylation score | TAC W4 | 0.05218 |
| Oxidative phosphorylation score | TAC W4 | 0.162247 |
| Oxidative phosphorylation score | TAC W4 | -0.06741 |
| Oxidative phosphorylation score | TAC W4 | -0.10398 |
| Oxidative phosphorylation score | TAC W4 | -0.01835 |
| Oxidative phosphorylation score | TAC W4 | -0.10721 |
| Oxidative phosphorylation score | TAC W4 | -0.07365 |
| Oxidative phosphorylation score | TAC W4 | 0.46127 |
| Oxidative phosphorylation score | TAC W4 | 0.099457 |
| Oxidative phosphorylation score | TAC W4 | -0.06049 |
| Oxidative phosphorylation score | TAC W4 | 0.421929 |
| Oxidative phosphorylation score | TAC W4 | -0.06555 |
| Oxidative phosphorylation score | TAC W4 | -0.39678 |
| Oxidative phosphorylation score | TAC W4 | 0.496489 |
| Oxidative phosphorylation score | TAC W4 | 0.380084 |
| Oxidative phosphorylation score | TAC W4 | 0.15274 |
| Oxidative phosphorylation score | TAC W4 | -0.34798 |
| Oxidative phosphorylation score | TAC W4 | -0.12216 |
| Oxidative phosphorylation score | TAC W4 | 0.0764 |
| Oxidative phosphorylation score | TAC W4 | -0.39084 |
| Oxidative phosphorylation score | TAC W4 | -0.09426 |
| Oxidative phosphorylation score | TAC W4 | 0.520611 |
| Oxidative phosphorylation score | TAC W4 | 0.464419 |
| Oxidative phosphorylation score | TAC W4 | -0.00878 |
| Oxidative phosphorylation score | TAC W4 | -0.0449 |
| Oxidative phosphorylation score | TAC W4 | 0.189481 |
| Oxidative phosphorylation score | TAC W4 | 0.361999 |
| Oxidative phosphorylation score | TAC W4 | -0.03919 |
| Oxidative phosphorylation score | TAC W4 | 0.516735 |
| Oxidative phosphorylation score | TAC W4 | -0.15869 |
| Oxidative phosphorylation score | TAC W4 | 0.4673 |
| Oxidative phosphorylation score | TAC W4 | 0.308607 |
| Oxidative phosphorylation score | TAC W4 | 0.367125 |
| Oxidative phosphorylation score | TAC W4 | 0.563157 |
| Oxidative phosphorylation score | TAC W4 | 0.345493 |
| Oxidative phosphorylation score | TAC W4 | 0.027848 |
| Oxidative phosphorylation score | TAC W4 | 0.280954 |
| Oxidative phosphorylation score | TAC W4 | -0.09608 |
| Oxidative phosphorylation score | TAC W4 | 0.175778 |
| Oxidative phosphorylation score | TAC W4 | 0.13642 |
| Oxidative phosphorylation score | TAC W4 | 0.254056 |
| Oxidative phosphorylation score | TAC W4 | 0.37141 |
| Oxidative phosphorylation score | TAC W4 | 0.487743 |
| Oxidative phosphorylation score | TAC W4 | 0.442332 |
| Oxidative phosphorylation score | TAC W4 | 0.593158 |
| Oxidative phosphorylation score | TAC W4 | 0.412317 |
| Oxidative phosphorylation score | TAC W4 | 0.438033 |
| Oxidative phosphorylation score | TAC W4 | 0.380987 |
| Oxidative phosphorylation score | TAC W4 | 0.423739 |
| Oxidative phosphorylation score | TAC W4 | 0.535733 |
| Oxidative phosphorylation score | TAC W4 | 0.40078 |
| Oxidative phosphorylation score | TAC W4 | 0.118832 |
| Oxidative phosphorylation score | TAC W4 | 0.271808 |
| Oxidative phosphorylation score | TAC W4 | 0.241406 |
| Oxidative phosphorylation score | TAC W4 | 0.220088 |
| Oxidative phosphorylation score | TAC W4 | 0.007561 |
| Oxidative phosphorylation score | TAC W4 | 0.516466 |
| Oxidative phosphorylation score | TAC W4 | 0.222068 |
| Oxidative phosphorylation score | TAC W4 | 0.456394 |
| Oxidative phosphorylation score | TAC W4 | 0.25705 |
| Oxidative phosphorylation score | TAC W4 | 0.128791 |
| Oxidative phosphorylation score | TAC W4 | 0.507633 |
| Oxidative phosphorylation score | TAC W4 | 0.270557 |
| Oxidative phosphorylation score | TAC W4 | 0.257348 |
| Oxidative phosphorylation score | TAC W4 | 0.316598 |
| Oxidative phosphorylation score | TAC W4 | 0.211369 |
| Oxidative phosphorylation score | TAC W4 | 0.481163 |
| Oxidative phosphorylation score | TAC W4 | 0.318619 |
| Oxidative phosphorylation score | TAC W4 | -0.11517 |
| Oxidative phosphorylation score | TAC W4 | 0.484435 |
| Oxidative phosphorylation score | TAC W4 | 0.43237 |
| Oxidative phosphorylation score | TAC W4 | 0.393046 |
| Oxidative phosphorylation score | TAC W4 | -0.35033 |
| Oxidative phosphorylation score | TAC W8 | 0.482019 |
| Oxidative phosphorylation score | TAC W8 | -0.39564 |
| Oxidative phosphorylation score | TAC W8 | -0.12461 |
| Oxidative phosphorylation score | TAC W8 | -0.07391 |
| Oxidative phosphorylation score | TAC W8 | 0.221478 |
| Oxidative phosphorylation score | TAC W8 | 0.383331 |
| Oxidative phosphorylation score | TAC W8 | 0.430096 |
| Oxidative phosphorylation score | TAC W8 | -0.58024 |
| Oxidative phosphorylation score | TAC W8 | -0.08583 |
| Oxidative phosphorylation score | TAC W8 | 0.221994 |
| Oxidative phosphorylation score | TAC W8 | 0.427062 |
| Oxidative phosphorylation score | TAC W8 | 0.560762 |
| Oxidative phosphorylation score | TAC W8 | -0.34078 |
| Oxidative phosphorylation score | TAC W8 | -0.024 |
| Oxidative phosphorylation score | TAC W8 | 0.428073 |
| Oxidative phosphorylation score | TAC W8 | -0.46266 |
| Oxidative phosphorylation score | TAC W8 | -0.02142 |
| Oxidative phosphorylation score | TAC W8 | 0.270408 |
| Oxidative phosphorylation score | TAC W8 | -0.24873 |
| Oxidative phosphorylation score | TAC W8 | -0.20891 |
| Oxidative phosphorylation score | TAC W8 | -0.61431 |
| Oxidative phosphorylation score | TAC W8 | 0.38519 |
| Oxidative phosphorylation score | TAC W8 | -0.08944 |
| Oxidative phosphorylation score | TAC W8 | 0.028367 |
| Oxidative phosphorylation score | TAC W8 | -0.45434 |
| Oxidative phosphorylation score | TAC W8 | 0.544907 |
| Oxidative phosphorylation score | TAC W8 | 0.073244 |
| Oxidative phosphorylation score | TAC W8 | 0.479125 |
| Oxidative phosphorylation score | TAC W8 | 0.075087 |
| Oxidative phosphorylation score | TAC W8 | -0.67445 |
| Oxidative phosphorylation score | TAC W8 | -0.59354 |
| Oxidative phosphorylation score | TAC W8 | -0.5042 |
| Oxidative phosphorylation score | TAC W8 | -0.52007 |
| Oxidative phosphorylation score | TAC W8 | -0.44139 |
| Oxidative phosphorylation score | TAC W8 | -0.23869 |
| Oxidative phosphorylation score | TAC W8 | -0.69586 |
| Oxidative phosphorylation score | TAC W8 | 0.132319 |
| Oxidative phosphorylation score | TAC W8 | 0.033412 |
| Oxidative phosphorylation score | TAC W8 | 0.445896 |
| Oxidative phosphorylation score | TAC W8 | 0.206894 |
| Oxidative phosphorylation score | TAC W8 | -0.25647 |
| Oxidative phosphorylation score | TAC W8 | -0.60529 |
| Oxidative phosphorylation score | TAC W8 | -0.53142 |
| Oxidative phosphorylation score | TAC W8 | 0.09787 |
| Oxidative phosphorylation score | TAC W8 | -0.38215 |
| Oxidative phosphorylation score | TAC W8 | -0.51776 |
| Oxidative phosphorylation score | TAC W8 | -0.70747 |
| Oxidative phosphorylation score | TAC W8 | -0.66967 |
| Oxidative phosphorylation score | TAC W8 | 0.053694 |
| Oxidative phosphorylation score | TAC W8 | -0.63764 |
| Oxidative phosphorylation score | TAC W8 | -0.32272 |
| Oxidative phosphorylation score | TAC W8 | -0.63036 |
| Oxidative phosphorylation score | TAC W8 | 0.371418 |
| Oxidative phosphorylation score | TAC W8 | -0.19548 |
| Oxidative phosphorylation score | TAC W8 | -0.15141 |
| Oxidative phosphorylation score | TAC W8 | -0.08191 |
| Oxidative phosphorylation score | TAC W8 | -0.25266 |
| Oxidative phosphorylation score | TAC W8 | -0.6889 |
| Oxidative phosphorylation score | TAC W8 | -0.26319 |
| Oxidative phosphorylation score | TAC W8 | -0.01102 |
| Oxidative phosphorylation score | TAC W8 | -0.22732 |
| Oxidative phosphorylation score | TAC W8 | -0.30041 |
| Oxidative phosphorylation score | TAC W8 | 0.324018 |
| Oxidative phosphorylation score | TAC W8 | -0.43676 |
| Oxidative phosphorylation score | TAC W8 | 0.405217 |
| Oxidative phosphorylation score | TAC W8 | -0.17234 |
| Oxidative phosphorylation score | TAC W8 | -0.57316 |
| Oxidative phosphorylation score | TAC W8 | 0.381607 |
| Oxidative phosphorylation score | TAC W8 | -0.12847 |
| Oxidative phosphorylation score | TAC W8 | -0.48348 |
| Oxidative phosphorylation score | TAC W8 | -0.57272 |
| Oxidative phosphorylation score | TAC W8 | 0.160029 |
| Oxidative phosphorylation score | TAC W8 | 0.022595 |
| Oxidative phosphorylation score | TAC W8 | -0.68653 |
| Oxidative phosphorylation score | TAC W8 | -0.66938 |
| Oxidative phosphorylation score | TAC W8 | -0.47111 |
| Oxidative phosphorylation score | TAC W8 | 0.15509 |
| Oxidative phosphorylation score | TAC W8 | -0.57372 |
| Oxidative phosphorylation score | TAC W8 | -0.16546 |
| Oxidative phosphorylation score | TAC W8 | -0.75736 |
| Oxidative phosphorylation score | TAC W8 | -0.68367 |
| Oxidative phosphorylation score | TAC W8 | 0.11643 |
| Oxidative phosphorylation score | TAC W8 | 0.113652 |
| Oxidative phosphorylation score | TAC W8 | -0.53463 |
| Oxidative phosphorylation score | TAC W8 | 0.166519 |
| Oxidative phosphorylation score | TAC W8 | -0.34204 |
| Oxidative phosphorylation score | TAC W8 | -0.47445 |
| Oxidative phosphorylation score | TAC W8 | -0.2238 |
| Oxidative phosphorylation score | TAC W8 | -0.63745 |
