# Supplementary material for: Ndufs1 Deficiency Aggravates the Mitochondrial Membrane Potential Dysfunction in Pressure Overload-Induced Myocardial Hypertrophy
Source: Oxid Med Cell Longev. 2021 Mar 3;2021:5545261. doi: 10.1155/2021/5545261 (PMC7952157; doi:10.1155/2021/5545261)
Supplement: Supplementary Materials — Additional file 1 Table S1: Gene Ontology enrichment terms for differentially expressed genes identified based on the scRNA-seq of pressure overload-induced myocardial hypertrophy. Additional file 2: Table S2: pathways correlated with pressure overload-induced myocardial hypertrophy based on time series-related GSEA. Additional file 3: Table S3: the oxidative phosphorylation score of single cardiomyocytes was calculated by GSVA. Additional file 4: Table S4: the cellular compartment-specific protein-protein interaction network of NDUFS1. Additional file 5: Table S5: biological process enrichment analyses of NDUFS1. [file 5545261.f1.zip › Table S4 The cellular compartment-specific protein-protein interaction network of NDUFS1.doc]

**Table S4 The cellular compartment-specific protein-protein interaction network of NDUFS1.**

| **source** | **target** | **Loc** | **score** |
| --- | --- | --- | --- |
| **NDUFS1** | NDUFA13 | Cytosol | 1 |
| **NDUFS1** | NDUFA13 | Mitochondrion | 1 |
| **NDUFS1** | NDUFA13 | Nucleus | 1 |
| **NDUFS1** | NDUFA13 | Extracellular | 1 |
| **NDUFS1** | NDUFA13 | Membrane | 1 |
| **NDUFS1** | ACADVL | Cytosol | 1 |
| **NDUFS1** | ACADVL | Mitochondrion | 1 |
| **NDUFS1** | ACADVL | Nucleus | 1 |
| **NDUFS1** | ACADVL | Extracellular | 1 |
| **NDUFS1** | ACADVL | Membrane | 1 |
| **NDUFS1** | HSPD1 | Membrane | 1 |
| **NDUFS1** | HSPD1 | Cytosol | 1 |
| **NDUFS1** | HSPD1 | Mitochondrion | 1 |
| **NDUFS1** | HSPD1 | Nucleus | 1 |
| **NDUFS1** | HSPD1 | Secretory-pathway | 1 |
| **NDUFS1** | HSPD1 | Extracellular | 1 |
| **NDUFS1** | HTRA2 | Cytosol | 1 |
| **NDUFS1** | HTRA2 | Mitochondrion | 1 |
| **NDUFS1** | HTRA2 | Nucleus | 1 |
| **NDUFS1** | HTRA2 | Secretory-pathway | 1 |
| **NDUFS1** | HTRA2 | Extracellular | 1 |
| **NDUFS1** | HTRA2 | Membrane | 1 |
| **NDUFS1** | TIMM23 | Mitochondrion | 1 |
| **NDUFS1** | TIMM23 | Extracellular | 1 |
| **NDUFS1** | TIMM23 | Membrane | 1 |
| **NDUFS1** | TIMM23 | Cytosol | 1 |
| **NDUFS1** | NDUFS3 | Membrane | 1 |
| **NDUFS1** | NDUFS3 | Mitochondrion | 1 |
| **NDUFS1** | NDUFS3 | Nucleus | 1 |
| **NDUFS1** | NDUFS3 | Extracellular | 1 |
| **NDUFS1** | CPT2 | Mitochondrion | 0.9915904 |
| **NDUFS1** | CPT2 | Nucleus | 0.9915904 |
| **NDUFS1** | CPT2 | Extracellular | 0.9915904 |
| **NDUFS1** | CPT2 | Membrane | 0.9915904 |
| **NDUFS1** | CPT2 | Cytosol | 0.9915904 |
| **NDUFS1** | NDUFV1 | Cytosol | 1 |
| **NDUFS1** | NDUFV1 | Mitochondrion | 1 |
| **NDUFS1** | NDUFV1 | Extracellular | 1 |
| **NDUFS1** | NDUFV1 | Membrane | 1 |
| **NDUFS1** | NDUFA9 | Mitochondrion | 1 |
| **NDUFS1** | NDUFA9 | Nucleus | 1 |
| **NDUFS1** | UQCRFS1 | Mitochondrion | 1 |
| **NDUFS1** | UQCRFS1 | Extracellular | 1 |
| **NDUFS1** | UQCRFS1 | Membrane | 1 |
| **NDUFS1** | UQCRFS1 | Cytosol | 1 |
| **NDUFS1** | NDUFC2 | Cytosol | 1 |
| **NDUFS1** | NDUFC2 | Mitochondrion | 1 |
| **NDUFS1** | NDUFC2 | Extracellular | 1 |
| **NDUFS1** | NDUFC2 | Membrane | 1 |
| **NDUFS1** | NDUFS2 | Mitochondrion | 1 |
| **NDUFS1** | NDUFS2 | Nucleus | 1 |
| **NDUFS1** | NDUFS2 | Extracellular | 1 |
| **NDUFS1** | NDUFS2 | Membrane | 1 |
| **NDUFS1** | ACAD9 | Mitochondrion | 1 |
| **NDUFS1** | ACAD9 | Nucleus | 1 |
| **NDUFS1** | ACAD9 | Membrane | 1 |
| **NDUFS1** | NDUFA4 | Cytosol | 1 |
| **NDUFS1** | NDUFA4 | Mitochondrion | 1 |
| **NDUFS1** | NDUFA4 | Extracellular | 1 |
| **NDUFS1** | NDUFA4 | Membrane | 1 |
| **NDUFS1** | AIFM1 | Cytosol | 0.999831808 |
| **NDUFS1** | AIFM1 | Mitochondrion | 0.999831808 |
| **NDUFS1** | AIFM1 | Nucleus | 0.999831808 |
| **NDUFS1** | AIFM1 | Extracellular | 0.999831808 |
| **NDUFS1** | AIFM1 | Membrane | 0.999831808 |
| **NDUFS1** | YME1L1 | Mitochondrion | 1 |
| **NDUFS1** | YME1L1 | Nucleus | 1 |
| **NDUFS1** | YME1L1 | Extracellular | 1 |
| **NDUFS1** | YME1L1 | Membrane | 1 |
| **NDUFS1** | COX4I1 | Mitochondrion | 1 |
| **NDUFS1** | COX4I1 | Nucleus | 1 |
| **NDUFS1** | COX4I1 | Extracellular | 1 |
| **NDUFS1** | COX4I1 | Membrane | 1 |
| **NDUFS1** | NDUFB5 | Mitochondrion | 0.99365968 |
| **NDUFS1** | NDUFB5 | Nucleus | 0.99365968 |
| **NDUFS1** | NDUFB5 | Extracellular | 0.99365968 |
| **NDUFS1** | NDUFB5 | Membrane | 0.99365968 |
| **NDUFS1** | NDUFA12 | Cytosol | 1 |
| **NDUFS1** | NDUFA12 | Mitochondrion | 1 |
| **NDUFS1** | NDUFA12 | Extracellular | 1 |
| **NDUFS1** | NDUFA12 | Membrane | 1 |
| **NDUFS1** | NDUFV3 | Cytosol | 1 |
| **NDUFS1** | NDUFV3 | Mitochondrion | 1 |
| **NDUFS1** | NDUFV3 | Nucleus | 1 |
| **NDUFS1** | NDUFV3 | Extracellular | 1 |
| **NDUFS1** | NDUFV3 | Membrane | 1 |
| **NDUFS1** | SLC25A6 | Mitochondrion | 0.999946916 |
| **NDUFS1** | SLC25A6 | Nucleus | 0.999946916 |
| **NDUFS1** | SLC25A6 | Cytosol | 0.999946916 |
| **NDUFS1** | SLC25A6 | Extracellular | 0.999946916 |
| **NDUFS1** | SLC25A6 | Membrane | 0.999946916 |
| **NDUFS1** | SLC25A10 | Mitochondrion | 0.995361 |
| **NDUFS1** | SLC25A10 | Nucleus | 0.995361 |
| **NDUFS1** | SLC25A10 | Extracellular | 0.995361 |
| **NDUFS1** | SLC25A10 | Membrane | 0.995361 |
| **NDUFS1** | MRPL12 | Cytosol | 1 |
| **NDUFS1** | MRPL12 | Mitochondrion | 1 |
| **NDUFS1** | HSCB | Cytosol | 0.998 |
| **NDUFS1** | HSCB | Mitochondrion | 0.998 |
| **NDUFS1** | HSCB | Nucleus | 0.998 |
| **NDUFS1** | NDUFV2 | Membrane | 1 |
| **NDUFS1** | NDUFV2 | Mitochondrion | 1 |
| **NDUFS1** | NDUFV2 | Extracellular | 1 |
| **NDUFS1** | NDUFB6 | Mitochondrion | 0.99124552 |
| **NDUFS1** | NDUFB6 | Nucleus | 0.99124552 |
| **NDUFS1** | NDUFB6 | Extracellular | 0.99124552 |
| **NDUFS1** | NDUFB6 | Membrane | 0.99124552 |
| **NDUFS1** | NDUFA7 | Mitochondrion | 1 |
| **NDUFS1** | NDUFA7 | Nucleus | 1 |
| **NDUFS1** | NDUFA7 | Extracellular | 1 |
| **NDUFS1** | ATP5PF | Secretory-pathway | 1 |
| **NDUFS1** | ATP5PF | Mitochondrion | 1 |
| **NDUFS1** | ATP5PF | Extracellular | 1 |
| **NDUFS1** | ATP5PF | Cytosol | 1 |
| **NDUFS1** | AFG3L2 | Mitochondrion | 1 |
| **NDUFS1** | AFG3L2 | Extracellular | 1 |
| **NDUFS1** | AFG3L2 | Membrane | 1 |
| **NDUFS1** | UQCC1 | Secretory-pathway | 0.999570246 |
| **NDUFS1** | UQCC1 | Cytosol | 0.999570246 |
| **NDUFS1** | UQCC1 | Mitochondrion | 0.999570246 |
| **NDUFS1** | UQCC1 | Nucleus | 0.999570246 |
| **NDUFS1** | UQCC1 | Extracellular | 0.999570246 |
| **NDUFS1** | UQCC1 | Membrane | 0.999570246 |
| **NDUFS1** | ECSIT | Cytosol | 0.987 |
| **NDUFS1** | ECSIT | Mitochondrion | 0.987 |
| **NDUFS1** | ECSIT | Nucleus | 0.987 |
| **NDUFS1** | TIMMDC1 | Mitochondrion | 0.990982886 |
| **NDUFS1** | TIMMDC1 | Nucleus | 0.990982886 |
| **NDUFS1** | TIMMDC1 | Extracellular | 0.990982886 |
| **NDUFS1** | TIMMDC1 | Membrane | 0.990982886 |
| **NDUFS1** | ABCE1 | Cytosol | 0.999492774 |
| **NDUFS1** | ABCE1 | Mitochondrion | 0.999492774 |
| **NDUFS1** | ABCE1 | Extracellular | 0.999492774 |
| **NDUFS1** | ABCE1 | Membrane | 0.999492774 |
| **NDUFS1** | NDUFS4 | Mitochondrion | 1 |
| **NDUFS1** | NDUFS4 | Extracellular | 1 |
| **NDUFS1** | NDUFS4 | Membrane | 1 |
| **NDUFS1** | ATP6V1D | Cytosol | 0.998452965 |
| **NDUFS1** | ATP6V1D | Mitochondrion | 0.998452965 |
| **NDUFS1** | ATP6V1D | Nucleus | 0.998452965 |
| **NDUFS1** | ATP6V1D | Membrane | 0.998452965 |
| **NDUFS1** | ATP6V1D | Extracellular | 0.998452965 |
| **NDUFS1** | MRPL58 | Mitochondrion | 1 |
| **NDUFS1** | MRPL58 | Nucleus | 1 |
| **NDUFS1** | NDUFAF1 | Cytosol | 0.9985984 |
| **NDUFS1** | NDUFAF1 | Mitochondrion | 0.9985984 |
| **NDUFS1** | NDUFAF1 | Membrane | 0.9985984 |
| **NDUFS1** | NDUFAF3 | Secretory-pathway | 0.92656 |
| **NDUFS1** | NDUFAF3 | Cytosol | 0.92656 |
| **NDUFS1** | NDUFAF3 | Mitochondrion | 0.92656 |
| **NDUFS1** | NDUFAF3 | Nucleus | 0.92656 |
| **NDUFS1** | NDUFAF3 | Extracellular | 0.92656 |
| **NDUFS1** | TXNRD2 | Cytosol | 0.899 |
| **NDUFS1** | TXNRD2 | Mitochondrion | 0.899 |
| **NDUFS1** | TSG101 | Membrane | 0.98281072 |
| **NDUFS1** | TSG101 | Cytosol | 0.98281072 |
| **NDUFS1** | TSG101 | Nucleus | 0.98281072 |
| **NDUFS1** | TSG101 | Secretory-pathway | 0.98281072 |
| **NDUFS1** | TSG101 | Extracellular | 0.98281072 |
| **NDUFS1** | HUWE1 | Cytosol | 0.94505056 |
| **NDUFS1** | HUWE1 | Nucleus | 0.94505056 |
| **NDUFS1** | HUWE1 | Secretory-pathway | 0.94505056 |
| **NDUFS1** | HUWE1 | Extracellular | 0.94505056 |
| **NDUFS1** | HUWE1 | Membrane | 0.94505056 |
| **NDUFS1** | NOD2 | Membrane | 0.987484672 |
| **NDUFS1** | NOD2 | Cytosol | 0.987484672 |
| **NDUFS1** | NOD2 | Mitochondrion | 0.987484672 |
| **NDUFS1** | NOD2 | Nucleus | 0.987484672 |
| **NDUFS1** | NOD2 | Secretory-pathway | 0.987484672 |
| **NDUFS1** | NOD2 | Extracellular | 0.987484672 |
| **NDUFS1** | AURKAIP1 | Cytosol | 0.972 |
| **NDUFS1** | AURKAIP1 | Mitochondrion | 0.972 |
| **NDUFS1** | AURKAIP1 | Nucleus | 0.972 |
| **NDUFS1** | NDUFAF4 | Cytosol | 0.99928 |
| **NDUFS1** | NDUFAF4 | Mitochondrion | 0.99928 |
| **NDUFS1** | NDUFAF4 | Extracellular | 0.99928 |
| **NDUFS1** | NDUFA2 | Mitochondrion | 0.99964 |
| **NDUFS1** | NDUFA2 | Extracellular | 0.99964 |
| **NDUFS1** | NDUFA6 | Mitochondrion | 0.99964 |
| **NDUFS1** | NDUFA6 | Extracellular | 0.99964 |
| **NDUFS1** | UBC | Membrane | 0.973518926 |
| **NDUFS1** | UBC | Cytosol | 0.973518926 |
| **NDUFS1** | UBC | Mitochondrion | 0.973518926 |
| **NDUFS1** | UBC | Nucleus | 0.973518926 |
| **NDUFS1** | UBC | Secretory-pathway | 0.973518926 |
| **NDUFS1** | UBC | Extracellular | 0.973518926 |
| **NDUFS1** | XRN1 | Cytosol | 0.98384536 |
| **NDUFS1** | XRN1 | Nucleus | 0.98384536 |
| **NDUFS1** | XRN1 | Membrane | 0.98384536 |
| **NDUFS1** | XRN1 | Extracellular | 0.98384536 |
| **NDUFS1** | SFXN1 | Mitochondrion | 0.999737366 |
| **NDUFS1** | SFXN1 | Extracellular | 0.999737366 |
| **NDUFS1** | SFXN1 | Membrane | 0.999737366 |
| **NDUFS1** | HTT | Cytosol | 0.946504 |
| **NDUFS1** | HTT | Nucleus | 0.946504 |
| **NDUFS1** | HTT | Secretory-pathway | 0.946504 |
| **NDUFS1** | HTT | Membrane | 0.946504 |
| **NDUFS1** | STAT3 | Cytosol | 0.829774 |
| **NDUFS1** | STAT3 | Mitochondrion | 0.829774 |
| **NDUFS1** | STAT3 | Nucleus | 0.829774 |
| **NDUFS1** | STAT3 | Membrane | 0.829774 |
| **NDUFS1** | NDUFS7 | Cytosol | 0.92226416 |
| **NDUFS1** | NDUFS7 | Mitochondrion | 0.92226416 |
| **NDUFS1** | NDUFS7 | Nucleus | 0.92226416 |
| **NDUFS1** | NDUFS7 | Membrane | 0.92226416 |
| **NDUFS1** | VAPA | Cytosol | 0.98384536 |
| **NDUFS1** | VAPA | Nucleus | 0.98384536 |
| **NDUFS1** | VAPA | Secretory-pathway | 0.98384536 |
| **NDUFS1** | VAPA | Extracellular | 0.98384536 |
| **NDUFS1** | VAPA | Membrane | 0.98384536 |
| **NDUFS1** | NDUFA3 | Mitochondrion | 0.992403791 |
| **NDUFS1** | NDUFA3 | Extracellular | 0.992403791 |
| **NDUFS1** | NDUFA3 | Membrane | 0.992403791 |
| **NDUFS1** | NDUFS6 | Mitochondrion | 0.991254016 |
| **NDUFS1** | NDUFS6 | Extracellular | 0.991254016 |
| **NDUFS1** | NDUFS6 | Membrane | 0.991254016 |
| **NDUFS1** | TMEM126B | Mitochondrion | 0.999299642 |
| **NDUFS1** | TMEM126B | Extracellular | 0.999299642 |
| **NDUFS1** | TMEM126B | Membrane | 0.999299642 |
| **NDUFS1** | KRAS | Cytosol | 0.988691752 |
| **NDUFS1** | KRAS | Mitochondrion | 0.988691752 |
| **NDUFS1** | KRAS | Nucleus | 0.988691752 |
| **NDUFS1** | KRAS | Secretory-pathway | 0.988691752 |
| **NDUFS1** | KRAS | Extracellular | 0.988691752 |
| **NDUFS1** | KRAS | Membrane | 0.988691752 |
| **NDUFS1** | DUSP1 | Cytosol | 0.7 |
| **NDUFS1** | DUSP1 | Mitochondrion | 0.7 |
| **NDUFS1** | DUSP1 | Nucleus | 0.7 |
| **NDUFS1** | NDUFA5 | Mitochondrion | 0.99856 |
| **NDUFS1** | NDUFA5 | Extracellular | 0.99856 |
| **NDUFS1** | NDUFS8 | Secretory-pathway | 0.998 |
| **NDUFS1** | NDUFS8 | Mitochondrion | 0.998 |
| **NDUFS1** | NDUFB3 | Mitochondrion | 0.998861918 |
| **NDUFS1** | NDUFB3 | Extracellular | 0.998861918 |
| **NDUFS1** | NDUFB3 | Membrane | 0.998861918 |
| **NDUFS1** | MGST3 | Cytosol | 0.93418264 |
| **NDUFS1** | MGST3 | Nucleus | 0.93418264 |
| **NDUFS1** | MGST3 | Secretory-pathway | 0.93418264 |
| **NDUFS1** | MGST3 | Extracellular | 0.93418264 |
| **NDUFS1** | MGST3 | Membrane | 0.93418264 |
| **NDUFS1** | TRIM25 | Cytosol | 0 |
| **NDUFS1** | TRIM25 | Nucleus | 0 |
| **NDUFS1** | NDUFS5 | Mitochondrion | 0.94492 |
| **NDUFS1** | NDUFS5 | Extracellular | 0.94492 |
| **NDUFS1** | KSR2 | Cytosol | 0.881416 |
| **NDUFS1** | KSR2 | Nucleus | 0.881416 |
| **NDUFS1** | KSR2 | Extracellular | 0.881416 |
| **NDUFS1** | KSR2 | Membrane | 0.881416 |
| **NDUFS1** | COQ9 | Cytosol | 0.987 |
| **NDUFS1** | COQ9 | Mitochondrion | 0.987 |
| **NDUFS1** | EXOSC6 | Cytosol | 0 |
| **NDUFS1** | EXOSC6 | Nucleus | 0 |
| **NDUFS1** | TRMT61B | Cytosol | 0.958 |
| **NDUFS1** | TRMT61B | Mitochondrion | 0.958 |
| **NDUFS1** | TRMT61B | Nucleus | 0.958 |
| **NDUFS1** | UBE3A | Cytosol | 0 |
| **NDUFS1** | UBE3A | Nucleus | 0 |
| **NDUFS1** | COX14 | Mitochondrion | 0.997548746 |
| **NDUFS1** | COX14 | Extracellular | 0.997548746 |
| **NDUFS1** | COX14 | Membrane | 0.997548746 |
| **NDUFS1** | LIG4 | Cytosol | 0 |
| **NDUFS1** | LIG4 | Nucleus | 0 |
| **NDUFS1** | GOLT1B | Cytosol | 0.95108176 |
| **NDUFS1** | GOLT1B | Nucleus | 0.95108176 |
| **NDUFS1** | GOLT1B | Secretory-pathway | 0.95108176 |
| **NDUFS1** | GOLT1B | Extracellular | 0.95108176 |
| **NDUFS1** | GOLT1B | Membrane | 0.95108176 |
| **NDUFS1** | ND4 | Mitochondrion | 0.996323118 |
| **NDUFS1** | ND4 | Extracellular | 0.996323118 |
| **NDUFS1** | ND4 | Membrane | 0.996323118 |
| **NDUFS1** | C15orf48 | Mitochondrion | 0.91 |
| **NDUFS1** | C15orf48 | Nucleus | 0.91 |
| **NDUFS1** | LNPEP | Cytosol | 0.980619184 |
| **NDUFS1** | LNPEP | Secretory-pathway | 0.980619184 |
| **NDUFS1** | LNPEP | Extracellular | 0.980619184 |
| **NDUFS1** | LNPEP | Membrane | 0.980619184 |
| **NDUFS1** | NIT1 | Secretory-pathway | 0.79 |
| **NDUFS1** | NIT1 | Cytosol | 0.79 |
| **NDUFS1** | NIT1 | Mitochondrion | 0.79 |
| **NDUFS1** | NIT1 | Nucleus | 0.79 |
| **NDUFS1** | COX6B1 | Secretory-pathway | 0.991 |
| **NDUFS1** | COX6B1 | Mitochondrion | 0.991 |
| **NDUFS1** | HSPB2 | Cytosol | 0 |
| **NDUFS1** | HSPB2 | Nucleus | 0 |
| **NDUFS1** | EPHA2 | Membrane | 0.98488 |
| **NDUFS1** | EPHA2 | Cytosol | 0.98488 |
| **NDUFS1** | EPHA2 | Nucleus | 0.98488 |
| **NDUFS1** | EPHA2 | Secretory-pathway | 0.98488 |
| **NDUFS1** | EPHA2 | Extracellular | 0.98488 |
| **NDUFS1** | SCLT1 | Cytosol | 0.82388 |
| **NDUFS1** | SCLT1 | Membrane | 0.82388 |
| **NDUFS1** | RTEL1 | Cytosol | 0 |
| **NDUFS1** | RTEL1 | Nucleus | 0 |
| **NDUFS1** | CANX | Membrane | 0.982699296 |
| **NDUFS1** | CANX | Cytosol | 0.982699296 |
| **NDUFS1** | CANX | Secretory-pathway | 0.982699296 |
| **NDUFS1** | CANX | Extracellular | 0.982699296 |
| **NDUFS1** | NDUFAF8 | Mitochondrion | 0.96 |
| **NDUFS1** | L3MBTL3 | Nucleus | 0 |
| **NDUFS1** | SLC22A12 | Membrane | 0.98488 |
| **NDUFS1** | SLC22A12 | Extracellular | 0.98488 |
| **NDUFS1** | SLC22A12 | Cytosol | 0.98488 |
| **NDUFS1** | MTMR12 | Cytosol | 0 |
| **NDUFS1** | CST1 | Secretory-pathway | 0.9140352 |
| **NDUFS1** | CST1 | Extracellular | 0.9140352 |
| **NDUFS1** | CST1 | Membrane | 0.9140352 |
