# Supplementary material for: Ndufs1 Deficiency Aggravates the Mitochondrial Membrane Potential Dysfunction in Pressure Overload-Induced Myocardial Hypertrophy
Source: Oxid Med Cell Longev. 2021 Mar 3;2021:5545261. doi: 10.1155/2021/5545261 (PMC7952157; doi:10.1155/2021/5545261)
Supplement: Supplementary Materials — Additional file 1 Table S1: Gene Ontology enrichment terms for differentially expressed genes identified based on the scRNA-seq of pressure overload-induced myocardial hypertrophy. Additional file 2: Table S2: pathways correlated with pressure overload-induced myocardial hypertrophy based on time series-related GSEA. Additional file 3: Table S3: the oxidative phosphorylation score of single cardiomyocytes was calculated by GSVA. Additional file 4: Table S4: the cellular compartment-specific protein-protein interaction network of NDUFS1. Additional file 5: Table S5: biological process enrichment analyses of NDUFS1. [file 5545261.f1.zip › Table S5 NDUFS1 biological processes enrichment analyses.doc]

**Table S5 NDUFS1 biological processes enrichment analyses**

| **ID** | **Name** | **p-value** | **q-value FDR B&H** |
| --- | --- | --- | --- |
| **GO:0006120** | mitochondrial electron transport, NADH to ubiquinone | 2.78E-03 | 2.92E-02 |
| **GO:0010257** | NADH dehydrogenase complex assembly | 3.23E-03 | 2.92E-02 |
| **GO:0032981** | mitochondrial respiratory chain complex I assembly | 3.23E-03 | 2.92E-02 |
| **GO:0051881** | regulation of mitochondrial membrane potential | 4.12E-03 | 2.92E-02 |
| **GO:0033108** | mitochondrial respiratory chain complex assembly | 4.81E-03 | 2.92E-02 |
| **GO:0042775** | mitochondrial ATP synthesis coupled electron transport | 4.96E-03 | 2.92E-02 |
| **GO:0042773** | ATP synthesis coupled electron transport | 5.01E-03 | 2.92E-02 |
| **GO:0022904** | respiratory electron transport chain | 6.20E-03 | 2.92E-02 |
| **GO:0008637** | apoptotic mitochondrial changes | 7.05E-03 | 2.92E-02 |
| **GO:0006119** | oxidative phosphorylation | 7.59E-03 | 2.92E-02 |
| **GO:0015986** | ATP synthesis coupled proton transport | 7.74E-03 | 2.92E-02 |
| **GO:0015985** | energy coupled proton transport, down electrochemical gradient | 7.74E-03 | 2.92E-02 |
| **GO:0006754** | ATP biosynthetic process | 8.98E-03 | 2.92E-02 |
| **GO:0009206** | purine ribonucleoside triphosphate biosynthetic process | 9.58E-03 | 2.92E-02 |
| **GO:0009145** | purine nucleoside triphosphate biosynthetic process | 9.63E-03 | 2.92E-02 |
| **GO:0009201** | ribonucleoside triphosphate biosynthetic process | 9.88E-03 | 2.92E-02 |
| **GO:0022900** | electron transport chain | 1.00E-02 | 2.92E-02 |
| **GO:0009205** | purine ribonucleoside triphosphate metabolic process | 1.02E-02 | 2.92E-02 |
| **GO:0045333** | cellular respiration | 1.04E-02 | 2.92E-02 |
| **GO:0009142** | nucleoside triphosphate biosynthetic process | 1.04E-02 | 2.92E-02 |
| **GO:0009199** | ribonucleoside triphosphate metabolic process | 1.06E-02 | 2.92E-02 |
| **GO:0009144** | purine nucleoside triphosphate metabolic process | 1.07E-02 | 2.92E-02 |
| **GO:0009141** | nucleoside triphosphate metabolic process | 1.20E-02 | 3.12E-02 |
| **GO:1902600** | proton transmembrane transport | 1.38E-02 | 3.16E-02 |
| **GO:0015980** | energy derivation by oxidation of organic compounds | 1.55E-02 | 3.16E-02 |
| **GO:0009152** | purine ribonucleotide biosynthetic process | 1.59E-02 | 3.16E-02 |
| **GO:0072593** | reactive oxygen species metabolic process | 1.59E-02 | 3.16E-02 |
| **GO:0006164** | purine nucleotide biosynthetic process | 1.62E-02 | 3.16E-02 |
| **GO:0046034** | ATP metabolic process | 1.63E-02 | 3.16E-02 |
| **GO:0009260** | ribonucleotide biosynthetic process | 1.65E-02 | 3.16E-02 |
| **GO:0072522** | purine-containing compound biosynthetic process | 1.68E-02 | 3.16E-02 |
| **GO:0046390** | ribose phosphate biosynthetic process | 1.68E-02 | 3.16E-02 |
| **GO:0009165** | nucleotide biosynthetic process | 2.10E-02 | 3.73E-02 |
| **GO:1901293** | nucleoside phosphate biosynthetic process | 2.11E-02 | 3.73E-02 |
| **GO:0042391** | regulation of membrane potential | 2.57E-02 | 4.25E-02 |
| **GO:0009150** | purine ribonucleotide metabolic process | 2.78E-02 | 4.25E-02 |
| **GO:0007005** | mitochondrion organization | 2.81E-02 | 4.25E-02 |
| **GO:0009259** | ribonucleotide metabolic process | 2.86E-02 | 4.25E-02 |
| **GO:0006091** | generation of precursor metabolites and energy | 2.88E-02 | 4.25E-02 |
| **GO:0006163** | purine nucleotide metabolic process | 2.88E-02 | 4.25E-02 |
| **GO:0019693** | ribose phosphate metabolic process | 2.90E-02 | 4.25E-02 |
| **GO:0072521** | purine-containing compound metabolic process | 3.05E-02 | 4.35E-02 |
| **GO:0009117** | nucleotide metabolic process | 3.45E-02 | 4.73E-02 |
| **GO:0006753** | nucleoside phosphate metabolic process | 3.48E-02 | 4.73E-02 |
| **GO:0015672** | monovalent inorganic cation transport | 3.54E-02 | 4.73E-02 |
| **GO:0090407** | organophosphate biosynthetic process | 3.73E-02 | 4.82E-02 |
| **GO:0017144** | drug metabolic process | 3.77E-02 | 4.82E-02 |
| **GO:0055086** | nucleobase-containing small molecule metabolic process | 3.92E-02 | 4.90E-02 |
